# Built and social environment characteristics associated with motorcyclist mortality in Latin American cities from the SALURBAL study

**DOI:** 10.1186/s40621-025-00611-y

**Published:** 2025-09-30

**Authors:** Ignacio Javier Yannone, Marcio Alazraqui, Jordan L. Rodriguez Hernandez, Olga Lucia Sarmiento Dueñas, Daniel A. Rodriguez, Carolina Pérez Ferrer, Luis A. Guzman, Mónica Serena Perner, Andrés Trotta, Ana V. Diez Roux, D. Alex Quistberg

**Affiliations:** 1 Instituto de Salud Colectiva, Universidad Nacional de Lanús, 29 de Septiembre 3901, B1832 Remedios de Escalada, Provincia de Buenos Aires, Argentina; 2 University of Washington, Seattle, WA USA; 3 Facultad de Medicina, Universidad de los Andes, Bogotá, Colombia; 4 Department of City and Regional Planning and Institute of Transportation Studies, University of California, Berkeley, CA USA; 5 Center for Research in Population Health, National Institute of Public Health, Cuernavaca, Morelos México; 6 Departamento de Ingeniería Civil y Ambiental Grupo de Sostenibilidad Urbana y Regional, Universidad de los Andes, Bogotá, Colombia; 7 Urban Health Collaborative, Dornsife School of Public Health, Drexel University, Philadelphia, PA USA

**Keywords:** Built environment, Cities, Latin america, Epidemiology, Traffic collisions, Motorcycles

## Abstract

**Background:**

Motorcyclists are the fastest growing road user group in Latin America, and account for 25% of all road traffic collision deaths. This study examines the relationship between motorcyclist mortality and the built and social urban environment in Latin American cities.

**Methods:**

We studied 337 cities with ≥ 100,000 inhabitants in seven Latin American countries. Mortality data from 2010 to 2019 were obtained from civil registries and linked to cities defined by the SALURBAL project. Motorcyclist deaths were identified using ICD-10 codes, with redistribution of ill-defined codes. City-level measures included population, urban development, street design, public transportation, and social environment. Associations were estimated using multilevel negative binomial models. A subanalysis of 300 cities with motorcycle registration data was conducted.

**Results:**

The crude city-level motorcyclist mortality rate was 4.16 per 100,000 population. Age-standardized rates varied from 0.51 to 22.60. Males had higher mortality rates, with the highest rates in 20-24-year-olds. After adjustment, cities with higher population density (RR 0.92 [95% CI 0.85–1.00]), intersection density (RR 0.91 [95% CI 0.83–0.99]), and social environment index (RR 0.88 [95% CI 0.83–0.93]) had lower motorcyclist mortality. More curvilinear street layout (RR 0.97 [95% CI 0.90,1.03]) and the presence of public transportation (RR 0.94 [95% CI 0.87,1.03]) showed a non-significant association with mortality. Higher urban development isolation (RR 1.07 [95% CI 1.00–1.14]) was associated with higher mortality, but the association weakened after adjustment. In cities with motorcycle registration data, higher rates of registered motorcycles were associated with higher motorcyclist mortality.

**Conclusion:**

Motorcyclist road traffic deaths in Latin American cities are associated with specific city-level characteristics. In fully adjusted models, higher intersection density and a stronger social environment index were linked to lower mortality rates. City-level interventions that improve street connectivity, promote safer and more cohesive urban environments, and address social inequities in infrastructure and services may help reduce motorcycle deaths and enhance road safety in the region.

**Supplementary Information:**

The online version contains supplementary material available at 10.1186/s40621-025-00611-y.

## Introduction

In 2019, motorcyclists were estimated to account for 30% of all traffic-related fatalities worldwide and 25% in the Americas [1]. Motorcycles have become an increasingly popular mobility option in cities across Latin America, and other low- and middle-income countries (LMICs) [2, 3], particularly since the beginning of the COVID-19 pandemic as delivery services that are reliant on two and three-wheeled motorized vehicles expanded [2]. This growth in motorcycle use has also been accompanied by rapid growth in motorcyclist road traffic injuries and deaths in the region [1, 2]. Young adult males are the primary users of motorcycles in Latin America, consequently representing the largest portion of fatalities, though female ridership is growing [1, 2]. In the last decade, the motorcycle industry in Latin America has experienced remarkable growth, increasing from 3.7 million units in 2012 to 5.6 million in 2023, which may worsen the increasing trends of motorcyclist injuries and deaths [4]. Notably, 2023 marked a new sales record, with a 4.6% increase compared to the previous year, highlighting the upward trend in demand for these vehicles in the region [4]. There has also been an increase in other two and three-wheeled motorized vehicles, such as scooters and e-bikes, that serve as intermediate vehicles between non-motorized pedal bicycles and motorized two-wheel vehicles [2]. Motorcycle use is perceived as advantageous, especially among households with limited resources, because of the lower cost of obtaining and operating a motorcycle compared to an automobile [2, 3]. Motorcycles provide increased mobility and access to employment opportunities given limited public transportation options and represent a fast and efficient alternative to traffic congestion compared to other transportation modes [2].

While preventive strategies such as helmet use, speed management, and alcohol and drug use prevention are well established to reduce injuries and deaths among motorcyclists [2], other contextual determinants are increasingly recognized as key to motorcyclist safety [2, 5]. Evidence suggests that characteristics of the built environment—such as road design, intersection density, traffic calming measures, and the presence of high-quality public transportation—can influence the risk and severity of motorcycle crashes [5–8]. At the same time, social inequalities play a major role: pedestrians, bicyclists, and motorcyclists are disproportionately affected by traffic injuries, with the heaviest burden often falling on the poorest and most vulnerable groups [1, 2, 9]. These inequities are observed not only between countries but also between cities [5] and within cities [9], where residents of lower-income neighborhoods are more exposed to hazardous traffic environments and have less access to safe infrastructure and emergency services.

Most existing research on motorcyclist road safety has primarily focused on individual-level risk factors—particularly helmet laws, their enforcement, and usage—which are key determinants of injury severity and fatalities [2, 10]. Other studies have examined the clinical outcomes of motorcycle crashes, describing the epidemiology of injuries, hospitalization rates, and survival [11, 12]. Traffic engineering research has largely concentrated on corridor- or intersection-level interventions aimed at high-risk crash locations, such as redesigning junctions or implementing localized traffic calming measures [13]. However, very few investigations have assessed how broader city-level characteristics—such as the spatial configuration of road networks, overall urban density, public transport availability, and socioeconomic conditions—may shape motorcyclist mortality risk. The limited evidence that does exist is derived mostly from studies of overall road traffic mortality rather than motorcyclist-specific outcomes [5–8, 14–17]. Recent multi-city analyses and reports have begun to highlight how features of urban design and transport infrastructure influence motorcycle crash patterns and deaths [18, 19]. Yet, dedicated city-level research on motorcyclist mortality remains scarce.

This study aims to address this gap by examining city-level motorcyclist deaths across Latin American cities in the decade preceding the COVID-19 pandemic and exploring their association with built and social environmental factors, to inform urban planning and public health interventions aimed at improving motorcyclist safety. Because many policies that influence the built environment, transportation systems, and road safety are formulated and implemented at the city level, focusing our analysis on this scale increases its relevance for policy and planning decisions.

## Methods

We conducted an ecological study of SALURBAL cities, defined as administrative units or clusters of administrative units encompassing the built-up areas of all urban agglomerations with ≥ 100,000 inhabitants as of 2010 in participating countries. The SALURBAL project is a multicenter initiative launched in 2017 aimed at compiling, harmonizing, and analyzing data on urban environments, social determinants, and health outcomes across Latin American cities to better understand the impacts of urbanization on population health [20]. The SALURBAL sample consists of 371 cities from 11 Latin American countries and integrates health outcomes together with built and social environment data to examine multilevel aspects of health in the region. For this study we focused on cities with sufficiently reliable and complete reporting of road traffic collision deaths (2010–2019), resulting in 337 cities across seven countries: 33 in Argentina, 152 in Brazil, 21 in Chile, 35 in Colombia, 1 in Costa Rica, 92 in Mexico, and 3 in Panama [20].

### Data sources

#### Mortality and population

Individual mortality data (2010–2019) were obtained from vital registration systems for each of the SALURBAL-defined cities. These data included information for location (e.g., municipality) of residence at death, sex, age, and the 10th revision of the International Classification of Diseases (ICD–10) cause of death. We defined motorcyclist road traffic deaths using ICD–10 codes V20–V39, which encompass deaths of both two-wheeled and three-wheeled motor vehicle occupants. We included all motorcycle rider deaths, whether road traffic-related or not, because although the proportion of non-traffic-related deaths is less than 1% of the total, it could still include deaths related to traffic incidents in parking lots, private roads, parks, etc. To address deaths coded with partially or ill-defined causes (approximately 11% of external cause deaths in all SALURBAL countries), we used well-established methods from prior studies [5, 21] to redistribute these codes across specifically-defined and partially-defined codes using 100 multinomial draws, based on the observed distribution of road-traffic deaths by age (5-year age groups), sex, and country, pooling periods of three years. This proportion varies across countries, and the redistribution algorithm accounts for such variability through country-specific adjustments.

We used official post-censal population projections and inter-censal estimations obtained from national census bureaus or equivalent sources for each country, harmonized for use in the SALURBAL project [20]. These data provided population counts by city, sex, and age group for each year from 2010 to 2019. To account for incomplete death registration, population projections were corrected using an ensemble of death distribution methods [20, 22], multiplying the population estimate for each year by a year-specific correction factor. We calculated mortality rates using total population-years, defined as the sum of the corrected yearly population estimates for each city across the study period. This approach incorporates temporal changes in population size, providing a more accurate basis for rate estimation than relying on a single population value for the entire decade.

#### Built and social environment variables

City-level exposures were selected a priori, based on a conceptual framework and empirical evidence linking urban form, street network configuration, and socioeconomic context to road traffic injury risk [5, 7, 13–15, 23, 24]. These variables capture structural and social features of cities that may shape patterns of mobility, crash risk, and injury severity.

The built environment characteristics investigated included features of the urban landscape, street design, transportation, and population density (Appendix pp.19–20). Urban landscape measures included patch density (density of separate built-up areas within city limits) as a measure of urban fragmentation and area-weighted mean nearest neighbor distance (how separated built-up areas are from one another on average) as measure of isolation of those areas. Street design variables included intersection density (number of intersections—defined as points where two or more streets converge—per square kilometer of built-up city area), street node average (i.e., average number of street segments radiating from each intersection), street length average, and circuity average (i.e., the average directness of road segments between intersections). Transportation variables encompassed the urban travel delay index (a measure of urban traffic congestion) and the presence or absence of mass transportation systems (rapid transit bus system or rail transit system). Population concentration was represented by population density for the year 2014 (population per square kilometer within the geographic boundaries of the urban built up area). In this analysis, we did not use the % city built-up as an urban environment variable because in previous SALURBAL studies a high positive correlation was found between this variable, intersection density (0.9), and patch density (0.7) [5].

Social environment was measured using the composite social environment index developed by the SALURBAL project [20], based on the most recent national census for each country (Argentina [2010], Brazil [2010], Chile [2002], Colombia [2005], Mexico [2010], Panama [2010], Costa Rica [2011]). This index comprises the proportion of households with piped water in their dwelling, the proportion of dwellings with a sewage network connection, the proportion of households with overcrowding (defined as more than three people per room; values for this component were reversed so that higher values indicate less overcrowding), and the proportion of adults aged 25 years or older who completed at least primary education (Appendix pp. 19–20). Higher index values indicate a better social environment.

We also included an estimate of Gross Domestic Product (GDP) per capita for each city, selecting the 2014 GDP value as it represents a midpoint of the 2010–2019 study period and aligns with the timing of other built environment variables in the dataset (Appendix pp.19–20). GDP estimates were obtained from harmonized global datasets compiled by Gennaioli et al. [25] and Kummu et al. [26], which model subnational economic output based on government, survey, and industry data and provide comparable gridded GDP values for 1990–2015. GDP was expressed in constant 2011 international US dollars, following the methodology of these datasets to ensure adjustment for inflation and purchasing power parity across countries. GDP was the only standardized economic indicator available for all 337 cities. Gross National Income (GNI) per capita was not available at the city level in most countries and primarily reflects national residents’ income, which may not accurately capture local economic production and resources accessible within individual cities.

GDP per capita was included as a standardized proxy for local economic activity and available resources, which can shape transport systems, infrastructure investment, enforcement capacity, and overall road safety conditions [1, 20, 23, 24]. Including this variable ensures that our models account for macro-level economic factors previously shown to be associated with road traffic mortality across countries and over time.

Finally, as a subanalysis for only those countries with information on vehicle registrations at the city level, we included as an urban environment variable the motorcycle motorization rate (motorcycle registrations per 1,000 inhabitants) calculated for the year 2014. Of the 337 cities included in our main analysis, 300 had this information available: 152 in Brazil, 21 in Chile, 35 in Colombia, and 92 in Mexico.

### Statistical analysis

We performed an exploratory data analysis of mortality rates across different age and sex groups among countries and cities using box plots and summary statistics. To do this, first we aggregated individual mortality data to construct mortality counts by country, city, sex, and 5-year age groups pooling the period 2010–2019 for each of the 100 redistributions. These death counts were linked to corresponding population counts for cities. We then averaged across the one hundred redistributions to estimate mortality rates by country, city, sex (both, males, or females) and age (all ages pooled together, or 17 categories of 5-years age groups). We also estimated an overall age-standardized mortality rate for each city using the direct standardization method and the World Health Organization (WHO) standard population data for 2000–2025 [27]. This standard is recommended for international comparative analyses of mortality as it reflects the projected global population structure for this period and ensures that differences in age distribution across cities do not bias mortality comparisons. It has been widely adopted in global road traffic safety reports [1, 28], in regional analyses for the Americas [29], and in recent multicountry studies of road traffic mortality in Latin American cities [5]. Using this standard allows direct comparability of our findings with prior international research and facilitates their interpretation within established global benchmarks. Additionally, we examined descriptive statistics of exposure variables by quartiles of city age-standardized rates and calculated Wald P values to examine differences between quartiles. For Brazil, Chile, Colombia, and Mexico, which had specific data on motorcycle registrations (including motorcycles, tricycles, and quadricycles) from 2010 to 2019, we also conducted an exploratory analysis of motorcyclist mortality rates using motorcycle registrations as the denominator. Since the registration data was not differentiated by sex or age group, we calculated city-specific mortality rates for both sexes and all age groups by averaging the mortality rates across the 100 redistributions.

We estimated rate ratios (RRs) of motorcyclist mortality associated with city features using a generalized linear model with negative binomial distribution and a random intercept for each city and 95% confidence intervals (95% CIs) with a robust variance estimator, and the corrected age-sex population counts as an offset. All the models were adjusted for country, sex (male or female) and age (5-year age groups). Each of the 100 redistributions was modelled separately, and coefficients and variances were extracted and then combined using Rubin’s rule to produce adjusted rate ratios (RRs) and 95% CIs. City-level exposures were standardized to a mean of 0 and a SD of 1 except for presence or absence of mass transit. We estimated two sets of models. Model 1 evaluated the association of each city-level exposure with motorcyclist mortality separately, adjusting for sex, age group, and country fixed effects. This model provides an adjusted effect estimate for each exposure considered individually. Model 2 was a multivariable model that included all city-level exposures simultaneously, while retaining adjustment for sex, age group, and country. This specification allowed us to estimate the independent association of each exposure with motorcyclist mortality, accounting for potential confounding and shared variance among the exposures. Additionally, for Brazil, Chile, Colombia, and Mexico, where information on the motorcycle motorization rate was available, we conducted a subanalysis incorporating this variable into both Models 1 and 2.

Our analyses include a set of variables that describe aspects of the spatial layout of streets in a city that are closely related (average street length, intersection density, average streets per node and average circuity). Because these variables are interrelated and may have collinear effects, we conducted a sensitivity analysis focusing on these characteristics (Appendix p. 16). This analysis allowed us to assess the robustness of our conclusions and determine how the interrelationships between these variables might influence the results, ensuring that the observed effects were consistent and not solely attributable to multicollinearity issues.

We used Stata (version 15.1) for descriptive and regression analyses, while the figures were created using ggplot2 in RStudio.

## Results

**Fig. 1:**
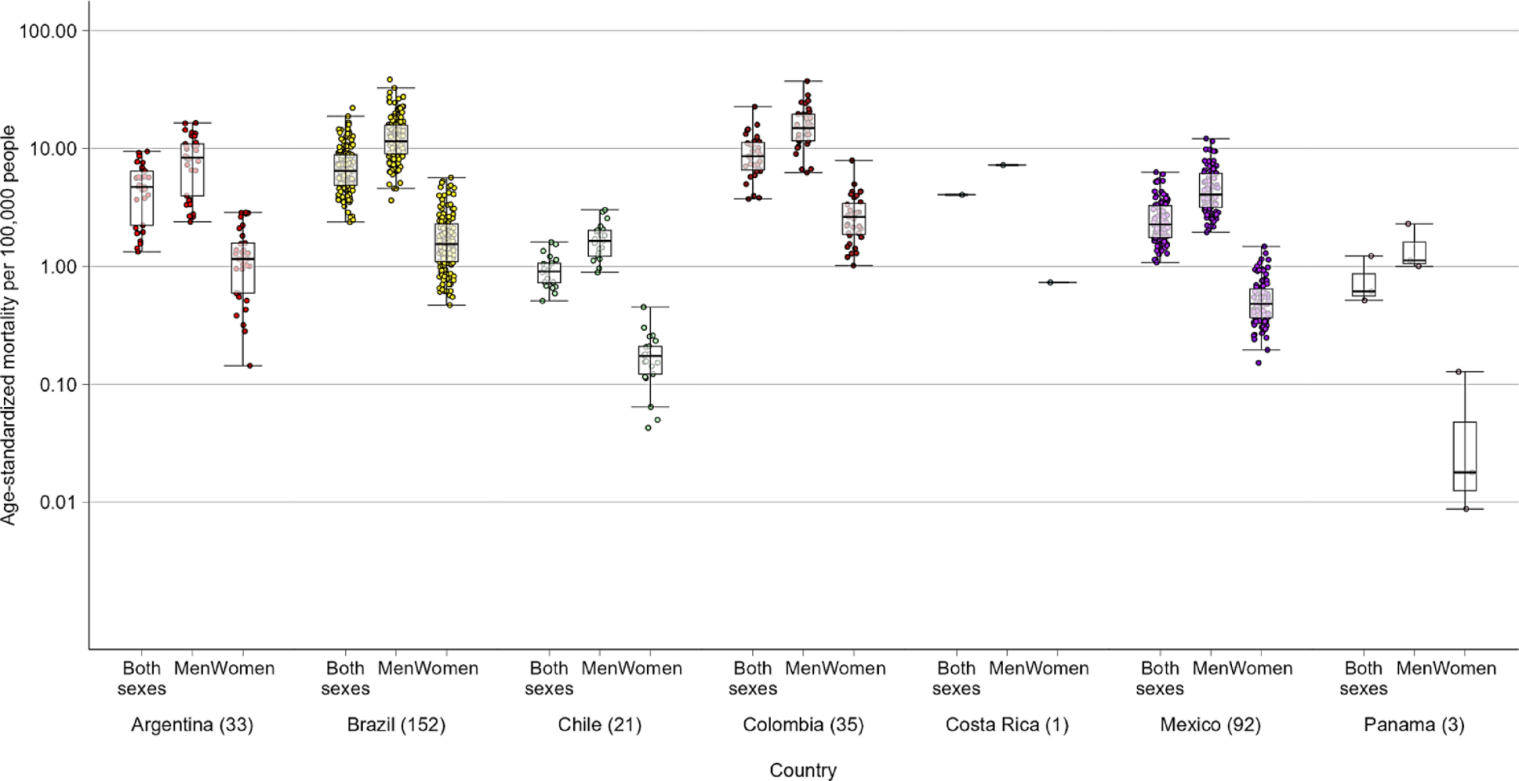
Age-standardized motorcyclist mortality rate per 100,000 population (2010–2019), by country and sex. The box represents the interquartile range (25th–75th percentile), and the line within the box represents the median

For the entire period (2010–2019) across 337 cities in Latin America there were 109,052 motorcyclist road traffic deaths among 2,620,000,000 population-year, resulting in a crude mortality rate of 4.16 per 100,000 population. Age-standardized rates varied substantially among countries and among cities within the countries (Table 1; Fig. 1).

At the country level, the median city-level age-standardized motorcyclist mortality rate ranged from a low of 0.61 per 100,000 population in Panama to a high of 8.60 per 100,000 population in Colombia (Table 1). Furthermore, substantial differences were observed in the median age-standardized mortality rates between males (1.12 to 14.89 per 100,000 population) and females (0.02 to 2.63 per 100,000 population) across the analyzed countries (Table 1).

**Table 1:** Median and interquartile range (IQR) city-level age-standardized motorcyclist mortality rates per 100,000 population by country and sex, 2010–2019 (*n* = 337)

Country	Cities (*n* = 337)	Median age-standardised mortality per 100.000 population of SALURBAL cities		
		Female Median (IQR)	Male Median (IQR)	Both Median (IQR)
Argentina	33	1.16 (0.59–1.58)	8.37 (3.98–10.91)	4.72 (2.23–6.43)
Brasil	152	1.54 (1.09–2.30)	11.51 (8.93–15.82)	6.46 (4.86–8.84)
Chile	21	0.17 (0.12–0.21)	1.64 (1.22–2.02)	0.91 (0.73–1.06)
Colombia	35	2.63 (1.83–3.51)	14.89 (11.55–19.59)	8.60 (6.50–11.45)
Costa Rica	1	0.73 (0.73–0.73)	7.23 (7.23–7.23)	4.05 (4.05–4.05)
Mexico	92	0.48 (0.37–0.65)	4.06 (3.17–6.14)	2.26 (1.75–3.29)
Panama	3	0.02 (0.01–0.13)	1.12 (1.00–2.29)	0.61 (0.51–1.23)

IQR (interquatile range)

Data are median (IQR) of mortality per 100,000 population

At the city-level, the age-standardized motorcyclist mortality rate ranged from 0.51 per 100,000 population in Punta Arenas, Chile to 22.60 per 100,000 population in Yopal, Colombia (Fig. 1; Appendix pp. 2–12). Differences in the age-standardized rates between males and females remained consistent across all cities, with rates being significantly higher in males (Fig. 1). The intraclass correlation coefficient for age-adjusted city-level motorcyclist mortality within countries was 0.11.

**Fig. 2:**
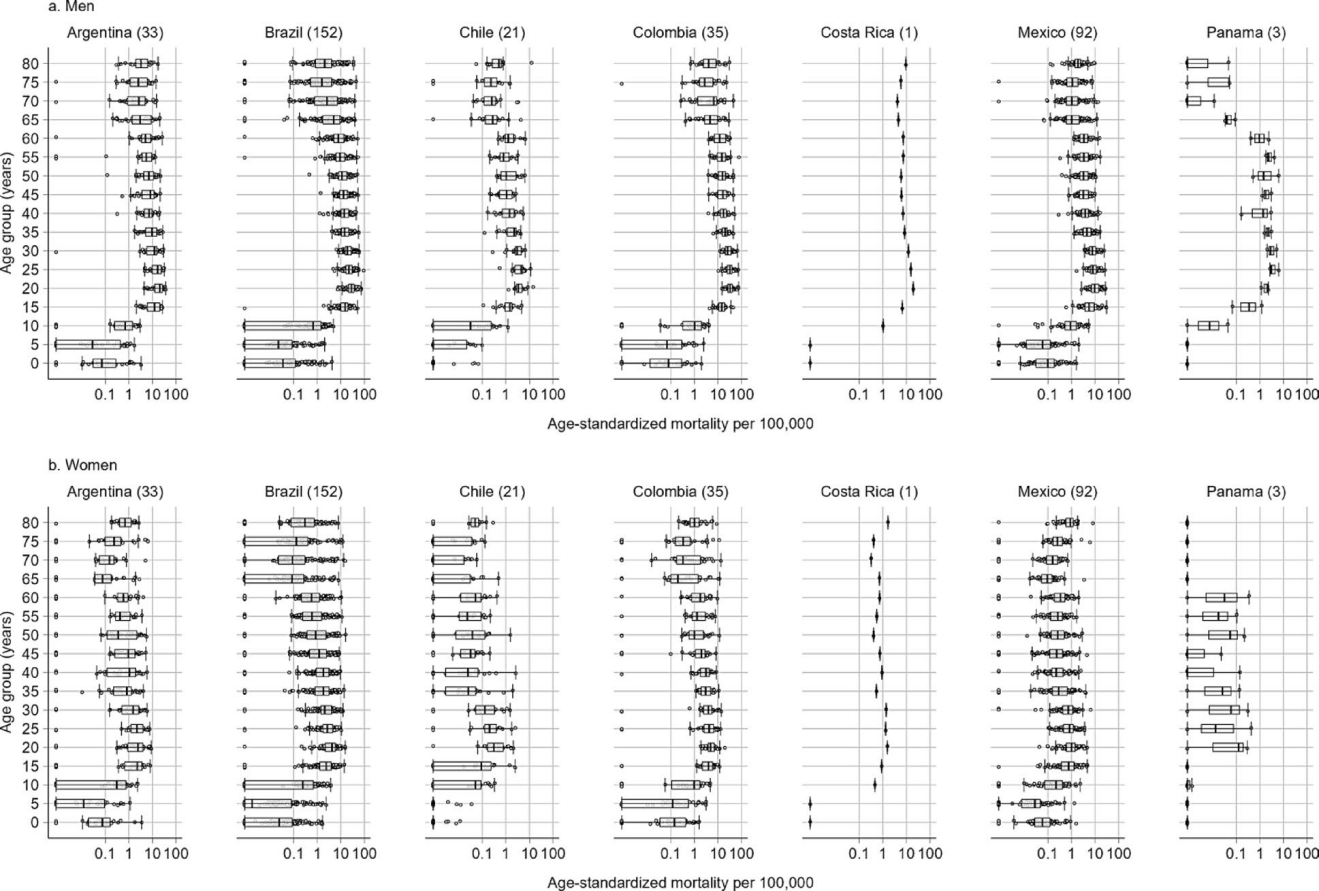
Boxplot of motorcyclist mortality rate per 100,000 population (2010–2019), by country, sex, and age group (A = Men, B = Women). The box represents the interquartile range (25th–75th percentile), and the line within the box represents the median

The motorcyclist mortality rate showed significant variability among age groups, ranging from 0 to 92.39 per 100,000 population. Additionally, it was noted that across all age groups, the motorcyclist mortality rate was consistently higher in men than in women (Fig. 2; Appendix pp. 13–14). The highest mortality rates were observed in the 20 to 24 age group for both males and females, with rates of 17.66 for males and 2.29 for females across 337 cities in Latin America (Fig. 2).

**Fig. 3:**
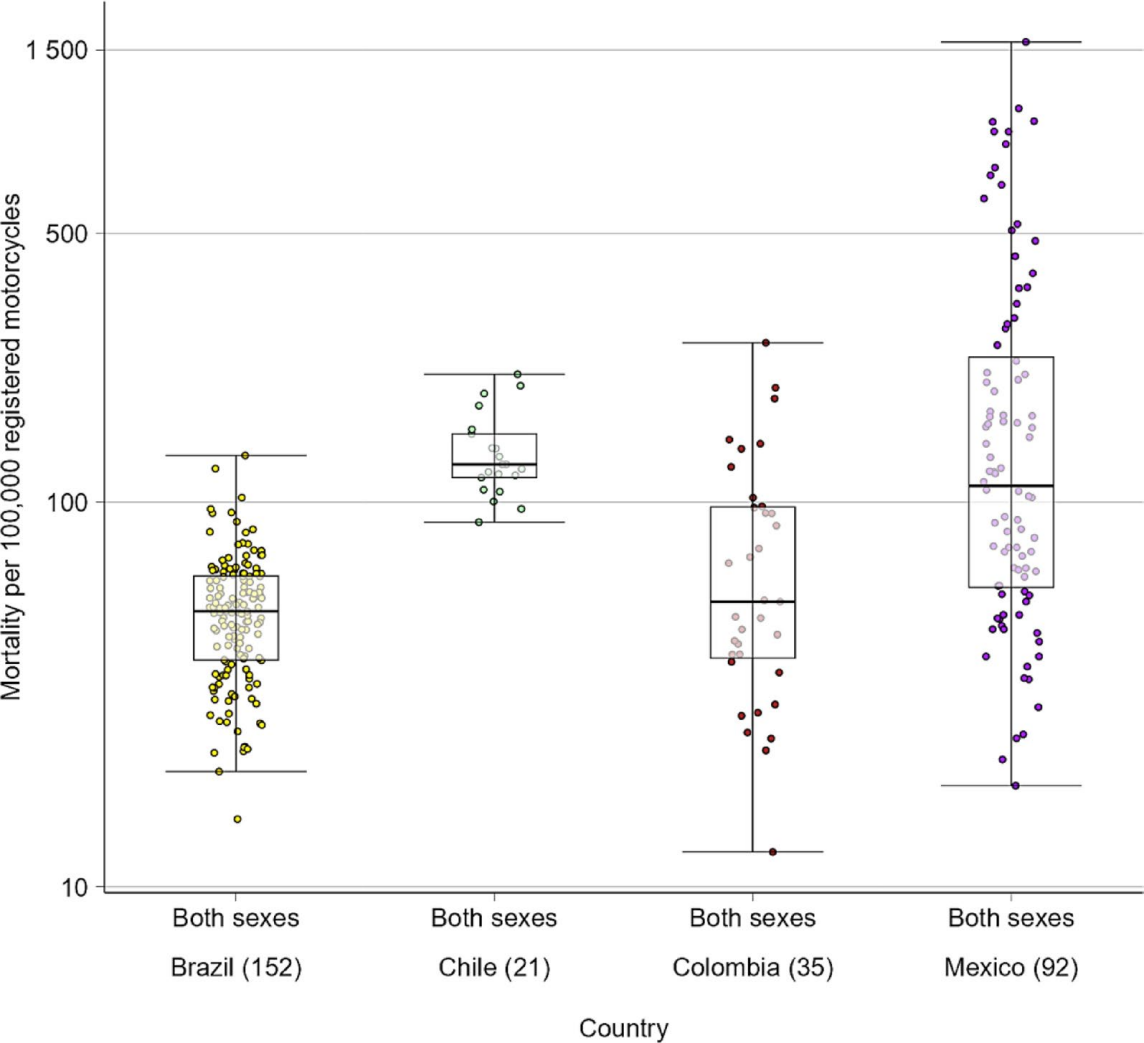
Motorcyclist mortality rate per 100,000 motorcycle registrations (2010–2019), by country. The box represents the interquartile range (25th–75th percentile), and the line within the box represents the median

In the countries where vehicle registration information was available (Brazil, Chile, Colombia and Mexico), the analysis of motorcycle registration mortality rates showed marked heterogeneity between countries and between cities within each country (Fig. 3). However, there were substantial differences compared to the mortality rates per 100,000 population in terms of which countries and cities had the highest and lowest values (Figs. 1 and 3). At the country level, median mortality rates by motorcycle registrations ranged from a low of 52.10 per 100,000 registrations in Brazil to a high of 125.31 per 100,000 registrations in Chile (Fig. 3). At the city level, mortality per 100,000 registrations ranged from 12.32 in Girardot in Colombia to 1,573.48 in Cuauhtémoc in Mexico (Fig. 3).

In descriptive analyses (Table 2), cities in the lowest per population mortality quartile had the highest social environment index (0.43 [−0.17 to 0.72]), the highest urban travel delay index (0.18 [0.11 to 0.26]), the lowest street length average (130.07 [115.41 to 153.97]), the lowest streets node average (2.93 [2.78 to 3.09]), and the highest urban development fragmentation (patch density 0.36 [0.12 to 0.61]). In contrast, cities in the highest mortality quartile had the lowest social environment index, (−0.17 [−0.45 to 0.24]), the lowest GDP per capita (10984 [7333 to 21098]), and the highest population density (7252.72 [5051.43 to 10863.37]). They also presented the greatest average distance between urbanized areas, hence greater urban isolation (88.56 [76.48 to 115.47]), the lowest patch density (0.20 [0.08 to 0.39]), the lowest intersection density (3.59 [1.53 to 6.71]), the highest number of streets per road node (3.06 [2.91 to 3.19]), the longest street length average, and the fewest number of cities with rail transit systems.

**Table 2:** City characteristics by quartiles of age-standardized motorcyclists mortality rates per 100.000 population

Exposure	Quartile 1 0.51 to 2.35 (*n* = 84)	Quartile 2 2.35 to 4.79 (*n* = 84)	Quartile 3 4.79 to 7.35 (*n* = 84)	Quartile 4 7.35 to 22.60 (*n* = 85)	Wald test *p* value****
Area-weighted Mean Nearest Neighbor Distance (meter)	81.25 (71.52 to 100.69)	84.84 (72.16 to 111.25)	78.18 (71.75 to 92.31)	88.56 (76.48 to 115.47)	0.0032
Patch density	0.36 (0.12 to 0.61)	0.35 (0.19 to 0.59)	0.35 (0.17 to 0.56)	0.20 (0.08 to 0.39)	0.0146
Circuity	1.07 (1.05 to 1.09)	1.06 (1.05 to 1.08)	1.07 (1.04 to 1.09)	1.06 (1.04 to 1.09	0.2785
Street length average	130.07 (115.41 to 153.97)	134.53 (118.75 to 164.55)	142.80 (121.33 to 172.48)	134.41 (121.71 to 165.88)	0.8969
Intersection Density	4.31 (1.62 to 10.54)	5.40 (2.17 to 9.26)	5.26 (3.00 to 10.62)	3.59 (1.53 to 6.71)	0.0048
Street Node Average	2.93 (2.78 to 3.09)	2.99 (2.88 to 3.13)	3.00 (2.86 to 3.16)	3.06 (2.91 to 3.19)	0.0674
Population density*	6291.31 (5361.47 to 7973.20)	6188.87 (5418.98 to 7807.30)	5746.57 (4857.74 to 7704.16)	7252.72 (5051.43 to 10863.37)	0.0032
Presence of bus rapid transit system or subway	13 (15.47%)	18 (21.43%)	14 (16.6%)	10 (11.76%)	0.0028
Urban travel delay index	0.18 (0.11 to 0.26)	0.11 (0.08 to 0.15)	0.09 (0.07 to 0.14)	0.10 (0.06 to 0.19)	0.0369
City gross domestic product per capita (US$)*	15,888 (11335 to 20169)	15,502 (11278 to 20413)	20,267 (11823 to 26038)	10,984 (7333 to 21098)	0.0003
Social environment index **	0.43 (−0.17 to 0.72)	0.37 (−0.13 to 0.53)	0.23 (−0.14 to 0.48	−0.17 (−0.45 to 0.24)	0.0116
Motorcycles registered per 1,000 population***	9.37 (5.51 to 18.62)	65.21 (25.39 to 106.83)	123.97 (92.90 to 168.057)	175.91 (133.56 to 212.57)	0.0441

*2014 data

** Argentina (2010); Brazil (2010); Chile (2002); Colombia (2005); Mexico (2010); Panama (2010); Costa Rica (2011). Higher values indicate a better social environment; overcrowding values were reversed for index calculation

*** 2014 data. Only Brazil, Chile, Colombia, Mexico

**** Test whether city characteristics differ across quartiles of age-standardized motorcyclists mortality

Although the city variables tended to be correlated with each other, the Pearson correlations (Appendix p. 15) were mostly weak (< 0.3) or moderate (0.3 to 0.6). The only strong correlation observed, i.e., greater than 0.6, was between patch density and intersection density (0.7).

**Fig. 4:**
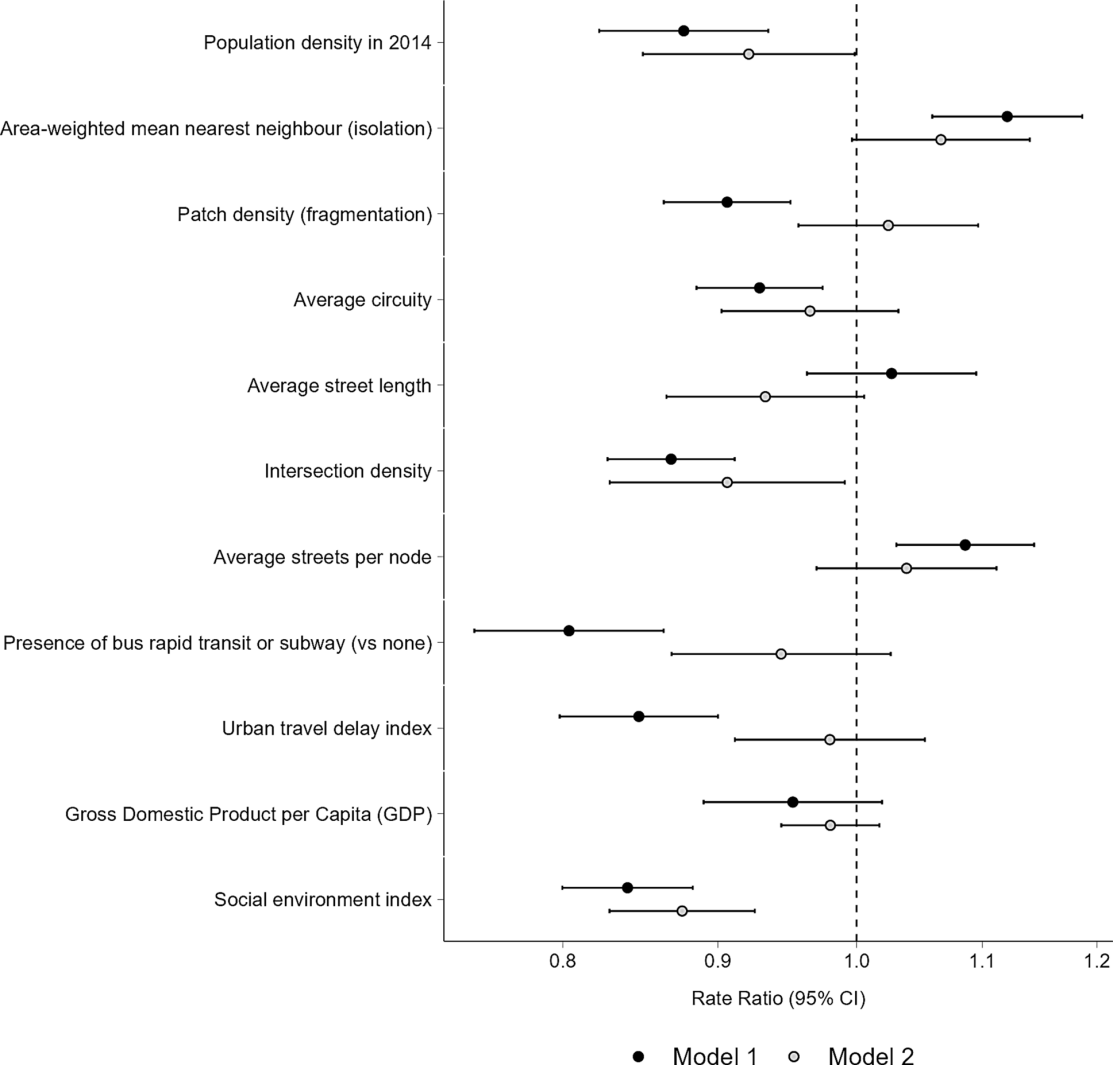
Rate ratios of motorcyclist mortality associated with city characteristics in single exposure (Model 1) and multivariable (Model 2) models. Rate ratios and 95% CI were estimated using a multilevel negative binomial model with random intercept for each city, robust variance estimator, and the corrected age-sex population counts as an offset. All the models were adjusted for country, sex (male or female) and age (5-year age groups). All RRs and 95% CIs reflect a difference of 1 SD except Presence bus rapid transit or subway. Model 1 is a single exposure model of each exposure. Model 2 includes all exposures in a multivariable model

In single-exposure models adjusted for age, sex and country fixed effects (Model 1), most variables related to the built and social environment of cities showed a significant association with motorcyclist mortality. Specifically, a higher social environment index (RR 0.84 [CI del 95% 0.80–0.88]), higher population density (RR 0.88 [95% CI 0.82–0.94]), and higher urban fragmentation (patch density) (RR 0.91 [95% CI 0.86–0.95]) were associated with lower motorcyclist mortality (Fig. 4). Similarly, a higher density of intersections (RR 0.87 [95% CI 0.83–0.91]), and a more curvilinear layout (i.e., with a higher circuity value) (RR 0.93 [95% CI 0.89–0.97]) were also associated with lower motorcyclist mortality (Fig. 4). The presence of a rapid transit bus or rail system was associated with lower motorcyclist mortality rates compared to those cities lacking these systems (RR 0.80 [95% CI 0.75–0.86]), and a higher urban travel delay index was also associated with lower mortality (RR 0.85 [95% CI 0.80–0.90]) (Fig. 4). Conversely, cities with greater isolation of urbanized areas (RR 1.12 [95% CI 1.06–1.19]) and higher average streets per node (RR 1.09 [95% CI 1.03–1.14]) were associated with higher motorcyclist mortality (Fig. 4). No associations were observed for average street length (RR 1.03 [95% CI 0.96–1.10]) and GDP per capita (RR 0.95 [95% CI 0.89–1.02]) in the single exposure model (Fig. 4).

In the multivariable model including all covariates simultaneously (Model 2), a higher social environment index (RR 0.88 [95% CI 0.83–0.93]), higher population density (RR 0.92 [95% CI 0.85,1.00]), and greater intersection density (RR 0.91 [95% CI 0.83–0.99]) continued to show a significant association with lower motorcyclist mortality (Fig. 4). The association with a more curvilinear street layout (RR 0.97 [95% CI 0.90–1.03]) and the presence of a rapid transit system (RR 0.94 [95% CI 0.87–1.03]) became weaker and their confidence intervals included the null (Fig. 4). Similarly, associations of a higher average streets per node (RR 1.04 [95% CI 0.97–1.11]), and higher isolation (RR 1.07 [95% CI 1.00–1.14]) with higher motorcyclist mortality were weakened and confidence intervals included the null after adjustment (Fig. 4). No associations were observed for the urban travel delay index (RR 0.98 [95% CI 0.91–1.05]) or patch density (RR1.02 [95% CI 0.96–1.10]) after multivariable adjustment (Fig. 4). For street length, the point estimate suggested a possible inverse association after multivariable adjustment (RR 0.93 [95% CI 0.87–1.01]); however, this association did not reach statistical significance in either Model 1 or Model 2 (Fig. 4). GDP was not associated with motorcyclist mortality after multivariable adjustment (RR 0.98 [95% CI 0.94–1.02]) (Fig. 4).

**Fig. 5:**
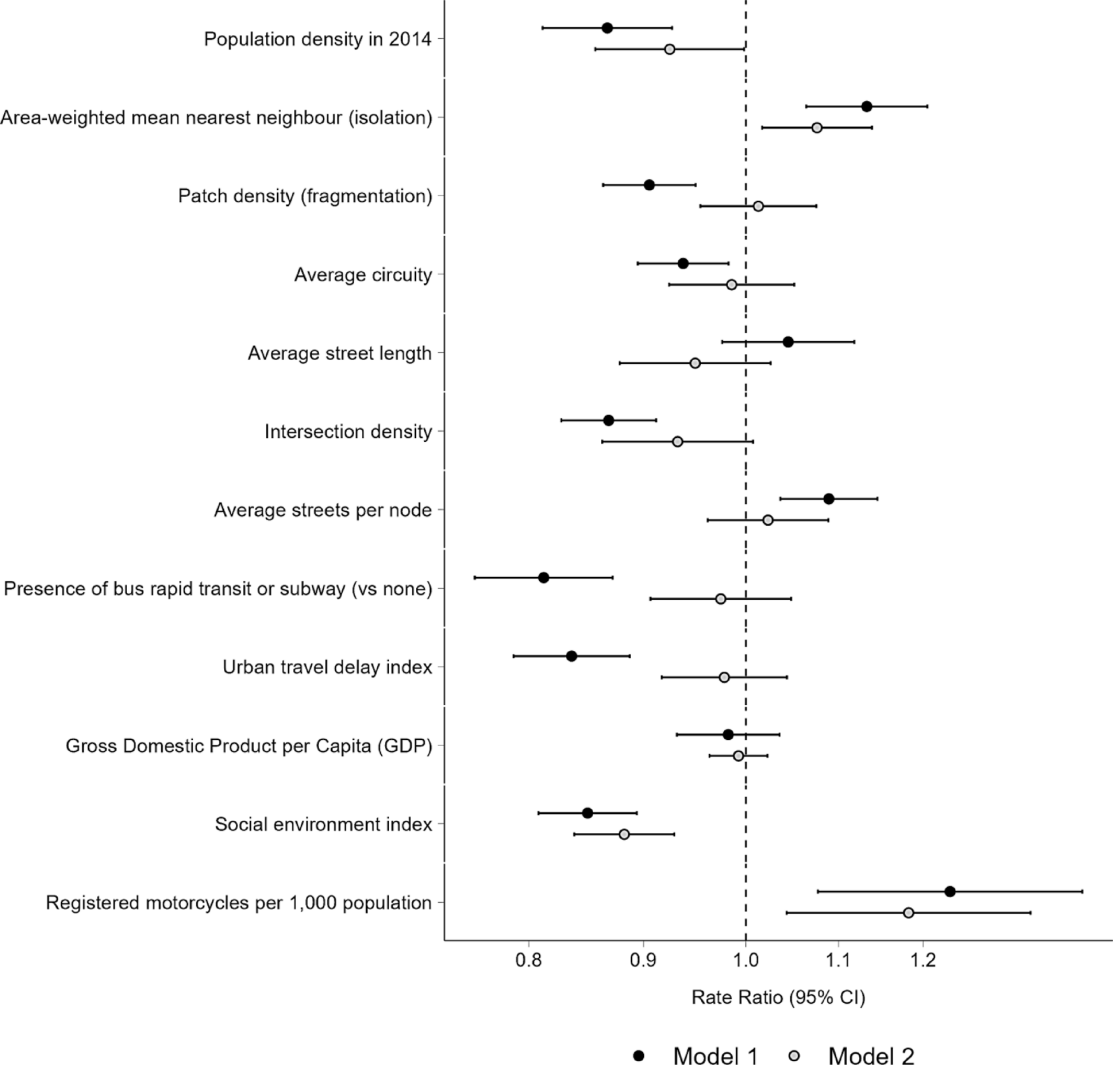
Rate ratios of motorcyclist mortality associated with city characteristics in single exposure (Model 1) and multivariable (Model 2) models including motorcycles registered per 1,000 population as a covariate. Subanalysis specific for Brazil, Chile, Colombia and Mexico. Rate ratios and 95% CI were estimated using a multilevel negative binomial model with random intercept for each city, robust variance estimator, and the corrected age-sex population counts as an offset. All the models were adjusted for country, sex (male or female) and age (5-year age groups). All RRs and 95% CIs reflect a difference of 1 SD except bus rapid transit or subway. Model 1 is a single exposure model of each exposure. Model 2 includes all exposures in a multivariable model

In the subanalysis in countries with motorcycle registration data (Brazil, Chile, Colombia and Mexico), a higher number of motorcycles registered per 1,000 population was associated with a higher mortality of motorcyclists due to traffic collisions (Fig. 5; RR 1.23 [95% CI 1.08–1.41]). For the other exposures included in models 1 and 2, the results show the multivariable model of the subanalysis presented very similar results to the corresponding model of the main analysis (Figs. 4 and 5), but, compared to the main analysis, higher isolation exhibited a stronger association (Figs. 4 and 5, RR 1.08 [95% CI 1.02–1.14]). In contrast, the association of higher intersection density was weakened (Figs. 4 and 5, RR 0.93 [95% CI 0.86–1.01]).

Finally, the sensitivity analyses, focused on improving model fit were consistent across all models in terms of the direction of the effect, with only minor variations in magnitude (Appendix p. 16).

## Discussion

In this study, we document that motorcyclist road traffic mortality is a major public health concern in 337 cities across seven Latin American countries, with age-adjusted mortality rates exceeding 50 deaths per 100,000 population annually in some cities, and young males being the most affected group. We also found marked heterogeneity across countries, with Colombian and Brazilian cities showing the highest mortality rates per 100,000 population, despite both having mandatory helmet laws, whereas Mexico lacked such a law during the 2010–2019 period [28]. These findings are consistent with prior research showing that helmet use laws, while effective in reducing injuries and fatalities [10–12], are not sufficient to fully prevent the burden of motorcycle deaths in rapidly motorizing settings [28].

Our findings suggest that motorcyclist mortality is closely linked to specific city-level characteristics. Cities with lower population density, weaker social environments, poor street connectivity, and more isolated development patterns tend to be secondary or tertiary cities that have expanded rapidly with limited planning and inadequate public transport infrastructure. In such contexts, motorcycles often emerge as an affordable and practical mobility option, compensating for the lack of reliable, safe, and accessible public transportation. In contrast, dense, well-connected cities with high intersection density and stronger social environments are more likely to support rail transit and safer mobility conditions. These results point to a potential public health benefit of compact, connected, and socially cohesive urban development strategies—traditionally promoted to encourage active mobility and public transit use—which may also contribute to reducing motorcycle-related fatalities [5, 7, 9, 14, 15, 23].

Our results indicate an increase in motorcyclist road deaths over recent years compared to earlier data [30]. Rodrigues et al. [30] found that, in the Americas, the overall motorcyclist mortality rate was 1.6 per 100,000 in the period 1998–2010. These rates have increased rapidly, ranging from 0.8 per 100,000 in 1998 to 3.5 per 100,000 in 2010 [30]. Our results also suggest that some of the Latin American countries in our study experience higher motorcyclist death rates than other LMICs. In particular, Argentina, Brazil, Colombia, and Costa Rica report motorcyclist mortality rates above the regional average for the Americas (3.6 per 100,000 inhabitants in 2016) [28]. Furthermore, Colombia and Brazil exceed the global average mortality rate of 5.1 per 100,000 inhabitants in 2016 [28].

The gap in mortality of motorcyclists between males and females and the 15–40 year-old age groups is notable and consistent with prior research [1–3, 28, 30]. We found that the median age-standardized mortality rates of males is about 7 to 10 times the rate of females, except in Panama where this difference is much more pronounced. Research suggests male motorcyclists tend to more frequently adopt risky behaviors, such as driving at high speeds, not wearing helmets, and driving under the influence of alcohol [2, 31]. In addition, the use of motorcycles is closely related to gender, being a means of transportation used predominantly by men, though recent trends suggest increasing adoption by women since the start of the COVID-19 pandemic [32]. For example, the results of mobility surveys conducted in different Latin American countries indicate that on average 78% of motorcycle users are men [32].

We also found significant heterogeneity in motorcyclist mortality across countries but also across cities within countries. This finding is similar to previous analyses of traffic-related deaths among all road users in the SALURBAL study where we also documented substantial between city heterogeneity [5]. Heterogeneity in motorcyclist mortality rates has been observed in other research focused on specific cities or regions within the same country [33]. Assessing sub-national and sub-regional heterogeneity is important for countries to understand and know where to direct resources to reducing and preventing motorcyclist mortality.

Marked disparities in motorcyclist mortality between specific cities and age groups likely reflect a combination of exposure, infrastructure, enforcement, and data factors. Differences in mortality per population—for example, between cities with high rates such as Yopal, Colombia (22.60 per 100,000 population) and low rates such as Punta Arenas, Chile (0.51 per 100,000)—may relate to varying levels of motorcycle use, patterns of use documented in prior regional analyses, road safety infrastructure, and quality of emergency response [2, 3, 13, 14]. When measured per registered motorcycle, extreme contrasts such as Cuauhtémoc, Mexico (1,573.48 per 100,000 registrations) versus Girardot, Colombia (12.32 per 100,000) may be influenced by differences in registration completeness, fleet size, and usage patterns, where smaller or irregularly registered fleets can inflate mortality rates [21]. Variability across age groups—with rates ranging from 0 to 92.39 per 100,000 population—is consistent with higher exposure and risk-taking behaviors among young adults, particularly males, who dominate motorcycle ridership in the region [11, 12]. These differences underscore that extreme values should be interpreted in light of local context, exposure measures, and data quality [1, 5, 11–14, 21].

In terms of city-level characteristics, while we found higher population density associated with lower motorcyclist road mortality, prior research has not been consistent, likely due to differences in the geographic level analyzed [5, 16, 34, 35]. Several mechanisms may explain the observed protective effect. First, denser urban areas typically provide greater visibility of vulnerable road users and have more traffic control infrastructure (e.g., traffic lights, marked crossings), which can help reduce collision risk [5, 7, 9, 13–15, 35, 36]. Second, traffic congestion in high-density areas is generally linked to lower average vehicle speeds, which reduces both the likelihood and severity of crashes, as supported by a recent meta-analytic synthesis [37]. Third, emergency medical response times are often shorter in dense urban areas, improving post-crash survival rates [1]. Finally, high-density cities frequently offer more accessible and extensive public transportation, reducing the need for motorcycle use for daily travel [5, 7, 16, 17]. Together, these factors may help explain the lower motorcyclist mortality observed in denser urban environments.

We found that greater isolation of urban development was associated with higher motorcyclist mortality, though it was weaker in multivariable analyses and stronger in the subanalysis including motorcycle registration. Cities with more isolated urban areas from one another (perhaps due to geography or historical urban development) may lead to a greater dependence on motorized vehicles (cars and motorcycles), particularly in the absence of high quality public transportation and may travel longer distances on high-speed, traffic-heavy roads to reach their destinations and with less connected streets, which could be associated with higher traffic fatalities [5, 38–40]. Motorcyclists typically are the road users with the highest risk of traffic collisions per mile traveled [40]. On the other hand, we found that greater fragmentation, i.e., higher patch density, was associated with lower motorcyclist mortality in traffic collisions in the single-exposure models, but was not associated in multivariable models.

Among street design metrics intersection density, a measure of connectivity, had the strongest association with higher values having lower motorcyclist road mortality. Previous research suggests that more connected cities have lower road traffic mortality [14, 15], potentially due to reduced motorized vehicle speeds, including motorcyclists, though this relationship may vary depending on local motorcycle traffic practices [8, 41]. More indirect streets (i.e., higher average circuity) were associated with lower motorcyclist mortality, although this association was non-significant in adjusted models, with confidence intervals including the null. Street directness is likely to affect motorcyclist speeding, visibility, and presence of safety barriers which have an impact on safety [42, 43]. These road design factors can be addressed via application of the Safe System to decrease motorized vehicle speeds in urban areas to 30 km per hour or less [1, 32]. Including motorcyclist in street planning will be key to addressing their injury and death risk in Latin American and other LMIC cities and not just considering automobile and cargo vehicle user needs.

Previous studies have shown inconsistent associations between congestion and road safety, as did our study. For example, Albalate and Fageda [44] found an inverted U-shaped relationship between congestion and road traffic collision deaths in 129 large European cities. Initially, congestion improves road safety, but beyond a certain point, it becomes counterproductive. This critical point is reached with a 30% increase in travel time compared to free-flow conditions. Another study conducted in Barcelona [45] focusing on motorcycle injuries highlighted that in situations of high congestion, injuries tended to be less severe or fatal, probably due to the reduction in speed in dense traffic, mitigating the severity of road traffic collisions.

While we expected cities with mass transit systems to have lower motorcyclist road mortality, we observed a non-significant association in multivariable models, with point estimates suggesting a potential protective effect but confidence intervals including the null. Prior research has suggested mixed effects on road safety following the implementation of measures for Bus Rapid Transit (BRT) systems, which decreased mortality in specific areas but increased it in others [6, 17]. It is hypothesized that mass transit systems such as BRT reduce collision and injuries by reducing speed on their routes, limiting traffic, and modifying surrounding infrastructure, though motorcyclists have not specifically been examined [17]. It may be that we observed weaker results in our study because we are not measuring the quality of the mass transit system, only the presence or absence. Additionally, we did not measure overall public transportation in cities, and the extent of mass transit systems varies substantially in the sample. Cities with more extensive, accessible public transportation may reduce the preference and uptake of motorcycle use, though other policy incentives may be needed in cities that already have high motorcycle use [32].

Cities with lower socioeconomic conditions exhibited higher mortality rates for motorcyclists. This may be explained by the fact that cities with higher economic development often have better road infrastructure, more connected street networks, and greater access to high-quality emergency and medical care, all of which contribute to safer environments for road users [5, 23, 24, 46]. In these cities, improved public transportation systems can reduce the reliance on motorcycles, while greater enforcement capacity and higher affordability of safety gear may increase helmet use and other protective behaviors [5, 17, 46]. We controlled for some of these infrastructure and policy characteristics, but still observed a strong association between the social environment index and motorcyclist road mortality, suggesting that other contextual or unmeasured social factors likely contribute. In contrast, we did not find an association between city-level GDP per capita and motorcyclist mortality. This finding differs from studies examining total road traffic mortality, which have shown that deaths often rise during periods of economic growth and decline during recessions, particularly in low- and middle-income countries [23, 24, 47]. These dynamics may not apply equally to all road users. In low-income settings, economic hardship has also been linked to higher-risk behaviors (e.g., speeding, riding without a helmet, or carrying multiple passengers), weak enforcement of traffic laws, and limited emergency response systems —all of which may disproportionately affect motorcyclists [46, 48]. These factors may help explain why a composite measure of the social environment was more strongly associated with mortality than GDP alone.

While we used harmonized data extracted at the city level, we employed an ecological study design; thus, inference is limited to city-level patterns rather than individual-level or smaller geographic units. Our analysis relied on population as the denominator instead of vehicle- or motorcycle-miles traveled or trips, meaning that the associations we observed could differ if detailed exposure data were available. Because we used residence of decedents rather than the location of collisions, population is likely a better denominator as it aligns more closely with the numerator. A sub-analysis conducted for those countries where city-level motorcycle registration information was available indicated that including this data did not substantially change the associations observed, supporting the use of population as an appropriate denominator for city-level analyses.

Additionally, misclassification of motorcyclist road-traffic deaths is a potential limitation; however, we applied the well-established redistribution methods to minimize potential bias from ill-defined and partially defined causes of death. The correction factors for incomplete death registration are imperfect at the city level and could lead to either underestimation or overestimation of mortality in some cities. The proportion of ill-defined deaths is not uniform across countries, which could differentially affect mortality estimates despite the use of country-specific redistribution methods. Improving mortality data registration and coding in the region remains an important priority.

Another important limitation of the study is that our exposure variables were time-invariant and measured closer to the end of the study period (2010–2019), meaning that changes in motorization patterns or urban infrastructure during this decade could have affected the associations we observed. For many exposures, however, major city-level changes would not be expected even over a decade, as these are long-term structural measures such as intersection density and urban isolation. Mass transit was among the exposures most likely to have changed, particularly as many cities expanded these systems during the study period, but this could only be addressed in future longitudinal analyses.

Additionally, some relevant city-level variables could not be incorporated due to data limitations. These include the motorcycle-per-person ratio, vehicle-per-person ratio, and their interaction, as harmonized registration data were not uniformly available across all cities. Likewise, the variable ‘year’ could not be included because annual exposure data were unavailable for many cities, and most measures remained time-invariant during 2010–2019. Furthermore, our study was not designed to analyze temporal trends in motorcyclist mortality but rather to assess associations with long-term, largely stable city-level characteristics. The omission of these variables may limit the precision of our estimates. Future longitudinal studies with harmonized vehicle registration data, their ratios and interactions, and time-varying measures of city-level characteristics are needed to refine these associations and better capture dynamic effects over time.

While these limitations should be acknowledged, an important strength of this study is the use of cities as the primary unit of analysis. This scale is particularly relevant because many decisions shaping the built environment, transportation systems, and road safety are made and implemented locally. By aligning our analyses with the scale at which policy action occurs, the findings provide actionable evidence for urban policymakers, planners, and public health authorities. This city-level perspective enhances the real-world applicability of the results and supports the development of locally tailored interventions to improve motorcyclist safety.

Motorcyclist road traffic crashes represent a significant public health challenge, particularly affecting the youngest and most vulnerable populations, and imposing high social and economic costs on societies, health systems, and affected countries. In this multi-city analysis of Latin American urban areas, we found that motorcyclist mortality is strongly associated with specific city-level characteristics. Cities with lower population density, weaker social development, poor street connectivity, and more isolated development patterns—often secondary or rapidly expanding cities with limited urban planning—showed higher mortality rates. These findings indicate that both the built and social urban environment contribute substantially to motorcyclist risk, beyond individual-level behaviors or enforcement measures traditionally emphasized in road safety policies.

Based on these findings, three key policy implications emerge. First, enhancing street connectivity and intersection density, combined with safe street designs such as protected crossings, roundabouts, and traffic-calming measures, can help reduce high-speed segments where severe motorcycle crashes are most likely to occur. Second, promoting compact, service-rich urban environments can shorten motorcycle trip distances and decrease exposure to hazardous traffic conditions. Third, addressing social inequalities that concentrate road hazards in disadvantaged neighborhoods—through targeted infrastructure investments, equitable access to emergency services, and stronger enforcement of road safety measures—could substantially reduce the disproportionate burden of motorcycle deaths in these settings. These city-level interventions, grounded in our findings, can complement behavioral and enforcement-based strategies, contributing to safer urban mobility for motorcyclists across the region. While improved public transport systems may also help reduce motorcycle dependence, our study did not explicitly analyze transit modes, and this remains an important hypothesis for future research.

Future studies should explore the mechanisms through which urban form and social environment influence motorcyclist mortality, including interactions with transport modes and traffic mix, and evaluate the effectiveness of city-level interventions aimed at reducing fatal motorcycle crashes. Policymakers, urban planners, and public health stakeholders in Latin America should collaborate to systematically integrate road safety objectives into urban development agendas, ensuring that connected and equitable cities become a foundation for sustainable and safe mobility.

## Supplementary Information

Supplementary Material 1

## Data Availability

The SALURBAL project welcomes queries from anyone interested in learning more about its dataset and potential access to data. Some of the data used in this study is freely available at https://data.lacurbanhealth.org/. To learn more about SALURBAL’s dataset, visit the SALURBAL project website (https://drexel.edu/lac/salurbal/overview/) or contact the project at salurbal@drexel.edu. After publication of this study, study protocols, data dictionaries, and requested study data may be made available to interested investigators after they have signed a data use agreement with SALURBAL and if their study proposal, developed in collaboration with SALURBAL investigators, is approved by the SALURBAL proposal and publications committee. Some data may not be available to external investigators because of data confidentiality agreements.
