# Supplementary material for: Built and social environment characteristics associated with motorcyclist mortality in Latin American cities from the SALURBAL study
Source: Inj Epidemiol. 2025 Sep 30;12:61. doi: 10.1186/s40621-025-00611-y (PMC12487543; doi:10.1186/s40621-025-00611-y)
Supplement: Supplementary file 1 — Supplementary Material 1 [file 40621_2025_611_MOESM1_ESM.docx]

**Built and social environment characteristics associated with motorcyclist mortality in Latin American Cities from the SALURBAL study**

**SUPPLEMENTARY MATERIAL**

**Table of contents**

**Appendix I.** Age–standardized motorcyclist mortality rate per 100,000 population by country. City level (n=337), 2010–2019

**Appendix II.** Female and male 5–year motorcyclist’ accident mortality rate per 100,000 population by age group and country. Medians and interquartile range (IQR) of cities, 2010–2019

**Appendix III.** Pearson correlation matrix of exposure variables

**Appendix IV.** Sensitivity analysis results

**Appendix V.** Akaike Information Criterion (AIC) and Bayesian Information Criterion (BIC) for each of our multivariable models (models 2 to 7)

**Appendix VI.** Descriptions of exposure measures and their interpretation

**Appendix VI.** Coefficient of models 1–2 (Main analysis and Subanalysis)

**Appendix I. Age–standardized motorcyclist mortality rate per 100,000 population by country. City level (n=337), 2010**–**2019.**

| **Argentina** |  |
| --- | --- |
| **Urban Area** | **Age–adjusted motorcyclist accident mortality per 100,000 population, 2010**–**2019** |
| Bahía Blanca | 1.92 |
| Mar del Plata | 2.23 |
| San Nicolás de los Arroyos | 4.72 |
| Tandil | 1.95 |
| Catamarca | 7.70 |
| Córdoba | 3.68 |
| Río Cuarto | 4.71 |
| Resistencia | 7.59 |
| Comodoro Rivadavia | 1.42 |
| Corrientes | 4.61 |
| Buenos Aires | 1.33 |
| Concordia | 5.62 |
| Paraná | 4.49 |
| Formosa | 9.20 |
| Jujuy | 5.62 |
| Santa Rosa | 4.88 |
| La Rioja | 5.68 |
| Mendoza | 3.78 |
| San Rafael | 6.64 |
| Posadas | 5.77 |
| Neuquén–Plottier–Cipolletti | 4.52 |
| San Carlos de Bariloche | 2.12 |
| Salta | 4.04 |
| Santiago del Estero | 9.43 |
| Rosario | 4.79 |
| Santa Fe | 7.73 |
| San Juan | 6.89 |
| San Luis | 6.43 |
| Villa Mercedes | 8.61 |
| San Miguel de Tucumán–Tafí Viejo | 5.74 |
| Rawson–Trelew | 1.63 |
| Río Gallegos | 1.56 |
| Zárate–Campana | 1.90 |

| **Brasil** |  |
| --- | --- |
| **Urban Area** | **Age–adjusted motorcyclist accident mortality per 100,000 population, 2010**–**2019** |
| Rio Branco | 6.82 |
| Arapiraca | 14.43 |
| Maceió | 5.36 |
| Macapá | 5.23 |
| Manaus | 4.76 |
| Alagoinhas | 5.53 |
| Barreiras | 4.02 |
| Feira de Santana | 3.50 |
| Ilhéus | 4.66 |
| Itabuna | 3.56 |
| Jequié | 9.96 |
| Porto Seguro | 6.56 |
| Salvador | 2.66 |
| Teixeira de Freitas | 6.84 |
| Vitória da Conquista | 7.19 |
| Fortaleza | 5.77 |
| Juazeiro do Norte | 9.57 |
| Sobral | 16.61 |
| Brasília | 3.80 |
| Cachoeiro de Itapemirim | 13.88 |
| Guarapari | 7.54 |
| Linhares | 8.78 |
| Vitória | 4.83 |
| Anápolis | 9.23 |
| Goiânia | 8.86 |
| Rio Verde | 8.90 |
| Caxias | 22.10 |
| Imperatriz | 12.67 |
| São Luís | 4.45 |
| Cuiabá | 9.53 |
| Rondonópolis | 14.68 |
| Campo Grande | 8.89 |
| Dourados | 12.47 |
| Araguari | 6.55 |
| Barbacena | 4.95 |
| Belo Horizonte | 4.00 |
| Conselheiro Lafaiete | 2.60 |
| Divinópolis | 4.31 |
| Governador Valadares | 7.67 |
| Ipatinga | 5.36 |
| Itabira | 4.90 |
| Juiz de Fora | 3.88 |
| Montes Claros | 2.87 |
| Passos | 4.45 |
| Patos de Minas | 7.85 |
| Poços de Caldas | 3.63 |
| Pouso Alegre | 2.37 |
| Sete Lagoas | 5.92 |
| Teófilo Otoni | 7.45 |
| Uberaba | 5.65 |
| Uberlândia | 5.68 |
| Varginha | 3.73 |
| Belém | 2.48 |
| Castanhal | 9.26 |
| Marabá | 12.76 |
| Parauapebas | 18.79 |
| Santarém | 8.87 |
| Campina Grande | 10.83 |
| João Pessoa | 7.02 |
| Apucarana | 8.73 |
| Arapongas | 8.89 |
| Cascavel | 8.27 |
| Curitiba | 4.58 |
| Foz do Iguaçu | 10.01 |
| Guarapuava | 6.40 |
| Londrina | 8.64 |
| Maringá | 9.10 |
| Paranaguá | 7.74 |
| Ponta Grossa | 5.00 |
| Toledo | 9.67 |
| Caruaru | 9.04 |
| Garanhuns | 10.76 |
| Petrolina | 11.61 |
| Recife | 4.96 |
| Vitória de Santo Antão | 7.97 |
| Parnaíba | 14.52 |
| Teresina | 13.64 |
| Angra dos Reis | 7.96 |
| Araruama | 6.87 |
| Cabo Frio | 6.38 |
| Campos dos Goytacazes | 11.80 |
| Macaé | 6.13 |
| Nova Friburgo | 5.95 |
| Petrópolis | 5.38 |
| Resende | 3.23 |
| Rio de Janeiro | 3.83 |
| Teresópolis | 8.68 |
| Volta Redonda | 4.08 |
| Mossoró | 10.84 |
| Natal | 4.52 |
| Caxias do Sul | 4.08 |
| Passo Fundo | 4.53 |
| Pelotas | 5.77 |
| Porto Alegre | 3.52 |
| Rio Grande | 6.25 |
| Santa Cruz do Sul | 6.79 |
| Santa Maria | 3.93 |
| Uruguaiana | 3.66 |
| Ji–Paraná | 7.69 |
| Porto Velho | 12.07 |
| Boa Vista | 13.31 |
| Balneário Camboriú | 7.56 |
| Blumenau | 10.34 |
| Brusque | 10.44 |
| Chapecó | 7.10 |
| Criciúma | 6.94 |
| Florianópolis | 6.40 |
| Itajaí | 10.30 |
| Jaraguá do Sul | 8.83 |
| Joinville | 6.77 |
| Lages | 6.44 |
| Araçatuba | 7.50 |
| Araraquara | 6.60 |
| Araras | 6.13 |
| Atibaia | 7.35 |
| Santos | 5.36 |
| Barretos | 4.57 |
| Bauru | 5.67 |
| Birigui | 6.61 |
| Botucatu | 5.59 |
| Bragança Paulista | 8.83 |
| Campinas | 5.47 |
| Catanduva | 7.47 |
| Franca | 5.37 |
| Guaratinguetá | 4.31 |
| Itapetininga | 5.39 |
| Jaú | 7.34 |
| Jundiaí | 5.88 |
| Limeira | 4.78 |
| Marília | 4.75 |
| Mogi Guaçu | 5.43 |
| Ourinhos | 5.55 |
| Piracicaba | 5.49 |
| Presidente Prudente | 4.48 |
| Ribeirão Preto | 6.47 |
| Rio Claro | 6.33 |
| São Carlos | 4.61 |
| São José do Rio Preto | 6.39 |
| São José dos Campos | 4.40 |
| São Paulo | 3.49 |
| Sertãozinho | 5.62 |
| Sorocaba | 5.60 |
| Tatuí | 6.66 |
| Taubaté | 3.66 |
| Aracaju | 6.54 |
| Araguaína | 15.96 |
| Palmas | 13.35 |
| Tubarão | 10.74 |
| Bento Gonçalves | 5.00 |
| Caraguatatuba | 7.30 |
| Parobé | 7.33 |
| Rio das Ostras | 5.86 |

| **Chile** |  |
| --- | --- |
| **Urban Area** | **Age–adjusted motorcyclist accident mortality per 100,000 population, 2010–2019** |
| Arica | 0.89 |
| Iquique | 1.14 |
| Antofagasta | 0.73 |
| Calama | 1.35 |
| Copiapó | 0.96 |
| La Serena–Coquimbo | 1.06 |
| Valparaíso–Viña del Mar | 0.68 |
| Quillota | 0.91 |
| San Antonio | 0.79 |
| Santiago de Chile | 0.99 |
| Rancagua | 1.05 |
| Talca | 1.03 |
| Curicó | 1.54 |
| Concepción | 0.67 |
| Chillán | 1.21 |
| Los Ángeles | 1.61 |
| Temuco | 0.73 |
| Valdivia | 0.65 |
| Osorno | 0.74 |
| Puerto Montt | 0.59 |
| Punta Arenas | 0.51 |

| **Colombia** |  |
| --- | --- |
|  |  |
| **Urban Area** | **Age–adjusted motorcyclist accident mortality per 100,000 population, 2010–2019** |
| Apartadó | 13.28 |
| Medellín | 6.64 |
| Barranquilla | 3.81 |
| Cartagena | 4.99 |
| Tunja | 5.93 |
| Manizales | 3.96 |
| Florencia | 11.09 |
| Yopal | 22.60 |
| Popayán | 8.60 |
| Valledupar | 10.14 |
| Quibdó | 8.82 |
| Montería | 7.21 |
| Bogotá | 3.73 |
| Neiva | 14.29 |
| Riohacha | 11.45 |
| Santa Marta | 8.37 |
| Villavicencio | 11.48 |
| Pasto | 7.39 |
| Cúcuta | 6.70 |
| Armenia | 7.38 |
| Pereira | 7.06 |
| Barrancabermeja | 15.89 |
| Bucaramanga | 6.44 |
| Sincelejo | 10.70 |
| Ibagué | 7.80 |
| Buenaventura | 10.73 |
| Cali | 8.60 |
| Cartago | 12.14 |
| Palmira | 12.61 |
| Tuluá | 9.50 |
| Duitama | 5.75 |
| Girardot | 9.51 |
| Fusagasugá | 6.50 |
| Buga | 14.58 |
| Sogamoso | 6.41 |

| **Costa Rica** |  |
| --- | --- |
| **Urban Area** | **Age–adjusted motorcyclist accident mortality per 100,000 population, 2010–2019** |
| San José | 4.05 |

| **México** |  |
| --- | --- |
| **Urban Area** | **Age–adjusted motorcyclist accident mortality per 100,000 population, 2010–2019** |
| Aguascalientes | 2.67 |
| Ensenada | 1.97 |
| Mexicali | 1.58 |
| Tijuana | 1.48 |
| La Paz | 1.81 |
| Campeche | 3.67 |
| Ciudad del Carmen | 3.05 |
| Chihuahua | 2.14 |
| Juárez | 1.52 |
| Cuauhtémoc | 4.30 |
| Delicias | 2.94 |
| Hidalgo del Parral | 3.75 |
| San Cristóbal de las Casas | 1.14 |
| Tapachula | 2.64 |
| Tuxtla Gutiérrez | 1.64 |
| Acuña | 1.27 |
| Monclova | 2.20 |
| Piedras Negras | 2.28 |
| Saltillo | 1.67 |
| Torreón | 3.33 |
| Colima | 2.95 |
| Manzanillo | 5.30 |
| Tecomán | 5.39 |
| Durango | 3.50 |
| Acapulco de Juárez | 1.44 |
| Chilpancingo | 3.98 |
| Iguala | 2.38 |
| Celaya | 2.74 |
| Guanajuato | 1.94 |
| Irapuato | 3.25 |
| León | 1.99 |
| Uriangato | 3.97 |
| Salamanca | 3.32 |
| San Francisco del Rincón | 4.32 |
| Pachuca de Soto | 1.64 |
| Tula de Allende | 2.23 |
| Tulancingo de Bravo | 1.93 |
| Guadalajara | 2.10 |
| Ocotlán | 6.29 |
| Puerto Vallarta | 2.35 |
| Ciudad de México | 1.36 |
| Tianguistenco | 1.85 |
| Toluca | 1.55 |
| La Piedad | 3.72 |
| Morelia | 1.94 |
| Uruapan | 2.94 |
| Zamora | 3.34 |
| Cuautla | 2.46 |
| Cuernavaca | 1.46 |
| Tepic | 3.57 |
| Monterrey | 1.43 |
| Oaxaca de Juárez | 1.81 |
| San Juan Bautista Tuxtepec | 3.12 |
| Santo Domingo Tehuantepec | 2.34 |
| Puebla de Zaragoza | 1.73 |
| Tehuacán | 2.29 |
| Teziutlán | 1.50 |
| Querétaro | 2.17 |
| San Juan del Río | 2.35 |
| Cancún | 1.56 |
| Chetumal | 3.46 |
| Playa del Carmen | 2.22 |
| Culiacán | 5.21 |
| Los Mochis | 3.79 |
| Mazatlán | 2.38 |
| Ciudad Valles | 3.36 |
| Río Verde | 6.05 |
| San Luis Potosí | 2.71 |
| Obregón | 2.63 |
| Guaymas | 2.45 |
| Hermosillo | 1.62 |
| Navojoa | 5.21 |
| Nogales | 2.25 |
| San Luis Río Colorado | 1.91 |
| Villahermosa | 3.84 |
| Victoria | 2.56 |
| Matamoros | 1.69 |
| Nuevo Laredo | 1.95 |
| Reynosa | 1.79 |
| Tampico | 1.76 |
| Tlaxcala | 2.06 |
| Acayucan | 3.05 |
| Coatzacoalcos | 1.48 |
| Córdoba | 1.14 |
| Minatitlán | 1.93 |
| Orizaba | 1.08 |
| Poza Rica de Hidalgo | 2.08 |
| Veracruz | 1.28 |
| Xalapa | 1.36 |
| Mérida | 2.32 |
| Fresnillo | 4.11 |
| Zacatecas | 2.13 |

| **Panamá** |  |
| --- | --- |
| **Urban Area** | **Age–adjusted motorcyclist accident mortality per 100,000 population, 2010–2019** |
| Panamá City | 0.61 |
| Colón | 0.52 |
| David | 1.23 |

**Appendix B. Female 5–year motorcyclist’ accident mortality rate per 100,000 population by age group and country. Medians and interquartile range (IQR) of cities**

| **Age group (years)** | **Argentina  N=33 Median (IQR)** | **Brasil  N=152 Median (IQR)** | **Chile N=21 Median (IQR)** | **Colombia N=35 Median (IQR)** | **Costa Rica N=1 Median (IQR)** | **México N=92 Median (IQR)** | **Panamá  N=3 Median (IQR)** |
| --- | --- | --- | --- | --- | --- | --- | --- |
| **0** | 0.08 (0.02–0.16) | 0.02 (0–0.09) | 0 (0–0) | 0.14 (0.03–0.44) | 0 (0–0) | 0.06 (0.03–0.13) | 0 (0–0) |
| **5** | 0.01 (0–0.09) | 0.00 (0–0.08) | 0 (0–0) | 0.12 (0–0.62) | 0 (0–0) | 0.03 (0.01–0.05) | 0 (0–0) |
| **10** | 0.31 (0–0.76) | 0.24 (0–0.68) | 0.05 (0–0.09) | 0.99 (0.06–1.79) | 0.46 (0.46–0.46) | 0.22 (0.07–0.42) | 0 (0–0) |
| **15** | 2.29 (0.68–3.59) | 2.41 (1.26–3.91) | 0.09 (0–0.24) | 3.97 (2.15–6.08) | 0.89 (0.89–0.89) | 0.77 (0.38–1.31) | 0 (0–0) |
| **20** | 2.47 (0.91–3.94) | 4.21 (2.18–5.81) | 0.31 (0.17–0.78) | 4.87 (3.81–7.10) | 1.56 (1.56–1.56) | 1.02 (0.61–1.44) | 0.12 (0–0.29) |
| **25** | 2.23 (1.26–4.01) | 2.64 (1.75–4.46) | 0.20 (0.13–0.39) | 4.27 (2.56–7.03) | 1.32 (1.32–1.32) | 0.82 (0.46–1.21) | 0.01 (0–0.43) |
| **30** | 1.53 (0.51–2.64) | 2.07 (1.44–4.01) | 0.13 (0.05–0.33) | 3.94 (2.40–5.60) | 1.38 (1.38–1.38) | 0.74 (0.43–1.14) | 0.06 (0–0.31) |
| **35** | 0.83 (0.23–1.28) | 1.81 (0.81–3.20) | 0.03 (0.00–0.05) | 3.03 (1.82–5.13) | 0.54 (0.54–0.54) | 0.29 (0.16–0.68) | 0.03 (0–0.14) |
| **40** | 1.07 (0.12–1.96) | 1.81 (0.90–3.09) | 0.02 (0.00–0.07) | 3.14 (1.72–4.44) | 0.92 (0.92–0.92) | 0.23 (0.15–0.44) | 0 (0–0.14) |
| **45** | 0.95 (0.26–1.84) | 1.19 (0.47–2.45) | 0.03 (0.01–0.05) | 2.00 (1.00–2.99) | 0.75 (0.75–0.75) | 0.24 (0.12–0.43) | 0 (0–0.02) |
| **50** | 0.36 (0.12–2.19) | 0.86 (0.36–2.33) | 0.04 (0.01–0.13) | 1.01 (0.58–2.48) | 0.40 (0.40–0.40) | 0.27 (0.14–0.50) | 0.05 (0–0.22) |
| **55** | 0.43 (0.28–1.13) | 0.59 (0.24–1.55) | 0.02 (0.01–0.09) | 1.30 (0.82–3.09) | 0.55 (0.55–0.55) | 0.27 (0.13–0.42) | 0.02 (0–0.10) |
| **60** | 0.59 (0.33–0.91) | 0.57 (0.21–1.24) | 0.05 (0.01–0.09) | 1.78 (0.75–2.96) | 0.73 (0.73–0.73) | 0.35 (0.19–0.54) | 0.03 (0–0.35) |
| **65** | 0.08 (0.04–0.18) | 0.08 (0–0.27) | 0 (0–0.03) | 0.20 (0.09–1.60) | 0.71 (0.71–0.71) | 0.10 (0.05–0.15) | 0 (0–0) |
| **70** | 0.15 (0.06–0.24) | 0.09 (0.02–0.30) | 0 (0–0.02) | 0.33 (0.18–2.02) | 0.32 (0.32–0.32) | 0.16 (0.09–0.26) | 0 (0–0) |
| **75** | 0.24 (0.10–0.46) | 0.13 (0–0.40) | 0 (0–0.04) | 0.33 (0.14–0.67) | 0.41 (0.41–0.41) | 0.26 (0.17–0.42) | 0 (0–0) |
| **>80** | 0.69 (0.41–1.26) | 0.30 (0.08–0.75) | 0.05 (0.03–0.07) | 1.01 (0.62–1.59) | 1.64 (1.64–1.64) | 0.89 (0.58–1.19) | 0 (0–0) |

**Male 5–year motorcyclist’ accident mortality rate per 100,000 population by age group and country. Medians and interquartile range (IQR) of cities**

| **Age group (years)** | **Argentina  N=33 Median (IQR)** | **Brasil  N=152 Median (IQR)** | **Chile N=21 Median (IQR)** | **Colombia N=35 Median (IQR)** | **Costa Rica N=1 Median (IQR)** | **México N=92 Median (IQR)** | **Panamá  N=3 Median (IQR)** |
| --- | --- | --- | --- | --- | --- | --- | --- |
| **0** | 0.07 (0.03–0.28) | 0.03 (0–0.12) | 0 (0–0) | 0.08 (0.01–0.30) | 0 (0–0) | 0.10 (0.03–0.19) | 0 (0–0) |
| **5** | 0.03 (0–0.44) | 0.02 (0–0.09) | 0 (0–0.02) | 0.07 (0–0.31) | 0 (0–0) | 0.06 (0.01–0.13) | 0 (0–0) |
| **10** | 0.70 (0.25–1.45) | 0.67 (0–1.45) | 0.03 (0–0.24) | 1.03 (0.31–2.04) | 1.03 (1.03–1.03) | 0.92 (0.53–1.62) | 0.01 (0–0.04) |
| **15** | 12.34 (6.31–20.58) | 15.09 (10.00–19.51) | 1.48 (0.92–1.81) | 14.61 (11.02–19.76) | 6.71 (6.71–6.71) | 5.61 (3.70–9.16) | 0.35 (0.07–1.19) |
| **20** | 20.82 (12.64–26.54) | 28.21 (21.46–36.46) | 3.42 (2.87–4.78) | 32.59 (26.06–41.33) | 19.49 (19.49–19.49) | 9.79 (7.22–14.51) | 2.08 (1.13–2.29) |
| **25** | 16.81 (9.87–23.82) | 21.98 (16.51–29.89) | 4.49 (2.44–5.21) | 32.02 (24.75–40.84) | 15.42 (15.42–15.42) | 8.15 (6.49–11.56) | 3.10 (2.68–6.21) |
| **30** | 12.23 (5.86–15.54) | 18.92 (15.51–27.11) | 3.00 (2.52–4.47) | 25.86 (19.41–36.71) | 12.15 (12.15–12.15) | 7.76 (5.95–10.88) | 2.83 (2.02–5.11) |
| **35** | 9.63 (5.14–15.49) | 14.52 (10.55–21.61) | 2.28 (1.15–2.74) | 20.29 (15.36–25.18) | 8.39 (8.39–8.39) | 4.53 (3.22–6.69) | 2.19 (1.57–3.10) |
| **40** | 7.26 (4.58–9.67) | 13.84 (10.09–21.36) | 1.44 (0.72–2.26) | 17.30 (12.51–22.96) | 7.16 (7.16–7.16) | 3.85 (2.74–5.25) | 1.37 (0.16–2.99) |
| **45** | 8.23 (3.63–11.83) | 12.92 (8.92–18.88) | 1.08 (0.53–1.85) | 16.41 (9.93–22.87) | 6.13 (6.13–6.13) | 3.22 (2.20–5.26) | 1.76 (1.26–3.06) |
| **50** | 7.13 (4.36–11.22) | 11.04 (8.05–18.02) | 1.04 (0.63–2.73) | 15.81 (9.88–20.65) | 5.94 (5.94–5.94) | 3.26 (2.29–5.09) | 1.44 (0.51–6.20) |
| **55** | 5.58 (3.65–9.39) | 9.17 (6.24–14.56) | 0.80 (0.57–1.36) | 14.75 (9.37–20.84) | 7.33 (7.33–7.33) | 3.38 (2.28–5.19) | 2.35 (1.79–4.09) |
| **60** | 5.11 (3.56–8.61) | 7.80 (5.08–13.41) | 1.26 (0.94–2.18) | 11.80 (7.02–21.65) | 7.29 (7.29–7.29) | 3.41 (2.32–5.00) | 0.94 (0.41–2.38) |
| **65** | 3.02 (1.40–8.97) | 4.93 (1.74–9.22) | 0.28 (0.14–0.42) | 4.62 (2.58–11.02) | 4.56 (4.56–4.56) | 1.03 (0.61–2.12) | 0.04 (0.03–0.09) |
| **70** | 2.69 (0.90–4.99) | 2.58 (0.78–7.12) | 0.25 (0.12–0.37) | 6.66 (1.29–9.01) | 4.13 (4.13–4.13) | 1.04 (0.60–1.90) | 0 (0–0.01) |
| **75** | 2.55 (1.15–6.15) | 1.58 (0.51–4.11) | 0.23 (0.12–0.42) | 2.73 (1.68–6.51) | 5.79 (5.79–5.79) | 1.10 (0.64–1.93) | 0.05 (0–0.05) |
| **>80** | 3.26 (2.00–6.37) | 2.07 (0.83–4.08) | 0.51 (0.27–0.65 | 4.05 (2.37–8.37) | 9.48 (9.48–9.48) | 1.91 (1.43–2.46) | 0 (0–0.05) |

**Appendix III. Pearson correlation matrix of exposure variables**

| **Exposure** | Area–weighted Mean Nearest Neighbor Distance (meter) | Patch Density | Circuity | Street Length average | Intersection Density | Street Node Average | Population density | Presence of bus rapid transit system or subway | Urban travel delay index | City gross domestic product (US$) | Social environment index |
| --- | --- | --- | --- | --- | --- | --- | --- | --- | --- | --- | --- |
| Area–weighted Mean Nearest Neighbor Distance (meter) | 1 |  |  |  |  |  |  |  |  |  |  |
| Patch Density | –0.0218 | 1 |  |  |  |  |  |  |  |  |  |
| Circuity | 0.4357 | –0.0802 | 1 |  |  |  |  |  |  |  |  |
| Street Length average | –0.2252 | –0.2773 | 0.2875 | 1 |  |  |  |  |  |  |  |
| Intersection Density | 0.1571 | 0.6799 | –0.211 | –0.4523 | 1 |  |  |  |  |  |  |
| Street Node Average | –0.2499 | –0.3872 | –0.5608 | 0.0123 | –0.1127 | 1 |  |  |  |  |  |
| Population density) | 0.1658 | 0.3121 | –0.0376 | –0.1865 | 0.4878 | –0.0982 | 1 |  |  |  |  |
| Presence of bus rapid transit system or subway | –0.2126 | –0.0401 | 0.0072 | 0.1802 | –0.0301 | 0.0275 | –0.0419 | 1 |  |  |  |
| Urban travel delay index | –0.0383 | 0.0988 | 0.032 | –0.1376 | 0.2361 | 0.0614 | 0.1581 | 0.2201 | 1 |  |  |
| City gross domestic product (US$) | 0.5457 | 0.1194 | 0.1327 | –0.3289 | 0.2903 | –0.1831 | 0.2818 | –0.0909 | 0.221 | 1 |  |
| Social environment index | –0.0416 | –0.0691 | –0.2205 | –0.1245 | 0.0089 | 0.1547 | 0.0222 | –0.1137 | –0.4925 | –0.2641 | 1 |

**Appendix IV.** **Sensitivity analysis results and Akaike Information Criterion (AIC) and Bayesian Information Criterion (BIC) for each of our multivariable models**

Our analyses include a set of variables that describe aspects of the spatial layout of streets in a city that are closely related. Therefore, we conducted a sensitivity analysis focusing on these interrelated characteristics. We introduced four additional models (Models 3 to 6), each excluding either average street length or average circuity while including either average street per node or intersection density. We calculated the Akaike Information Criterion (AIC) and Bayesian Information Criterion (BIC) for each of our multivariable models (models 2 to 6). These were estimated for every one of the 100 iterations used in each model. The values were then compared against those of our saturated model (model 2). For each iteration of every model, we subtracted the corresponding value from that of model 2.


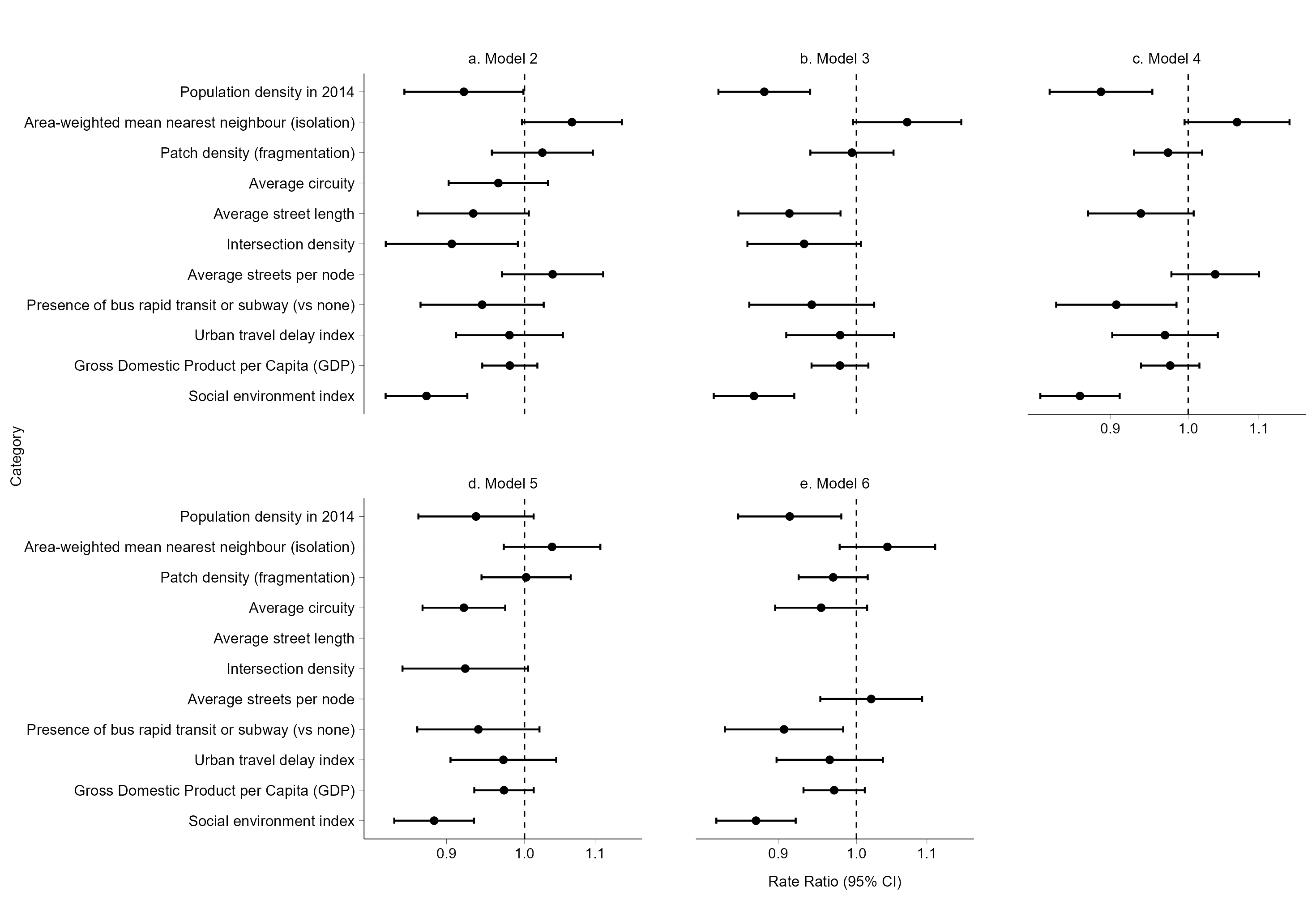


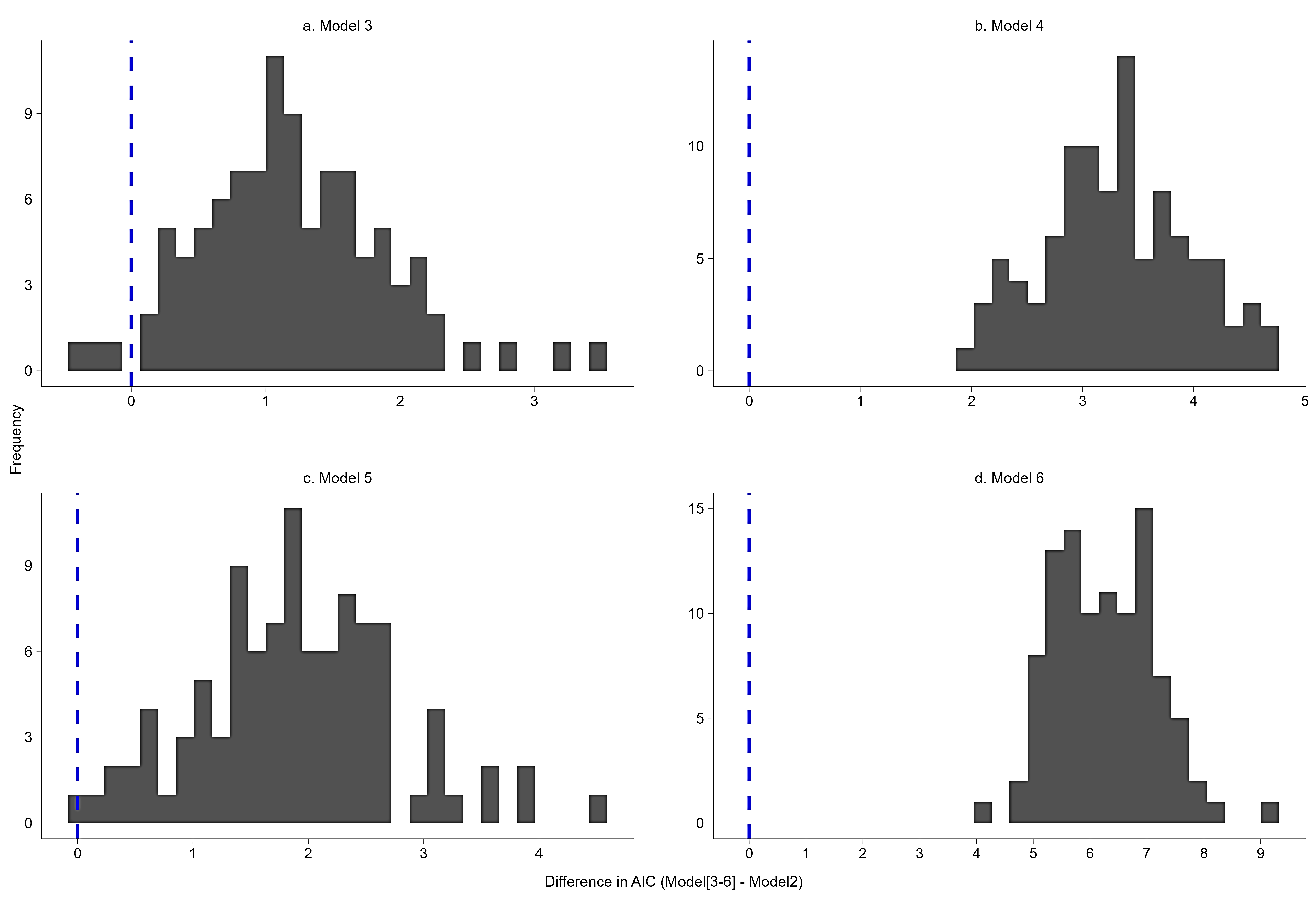


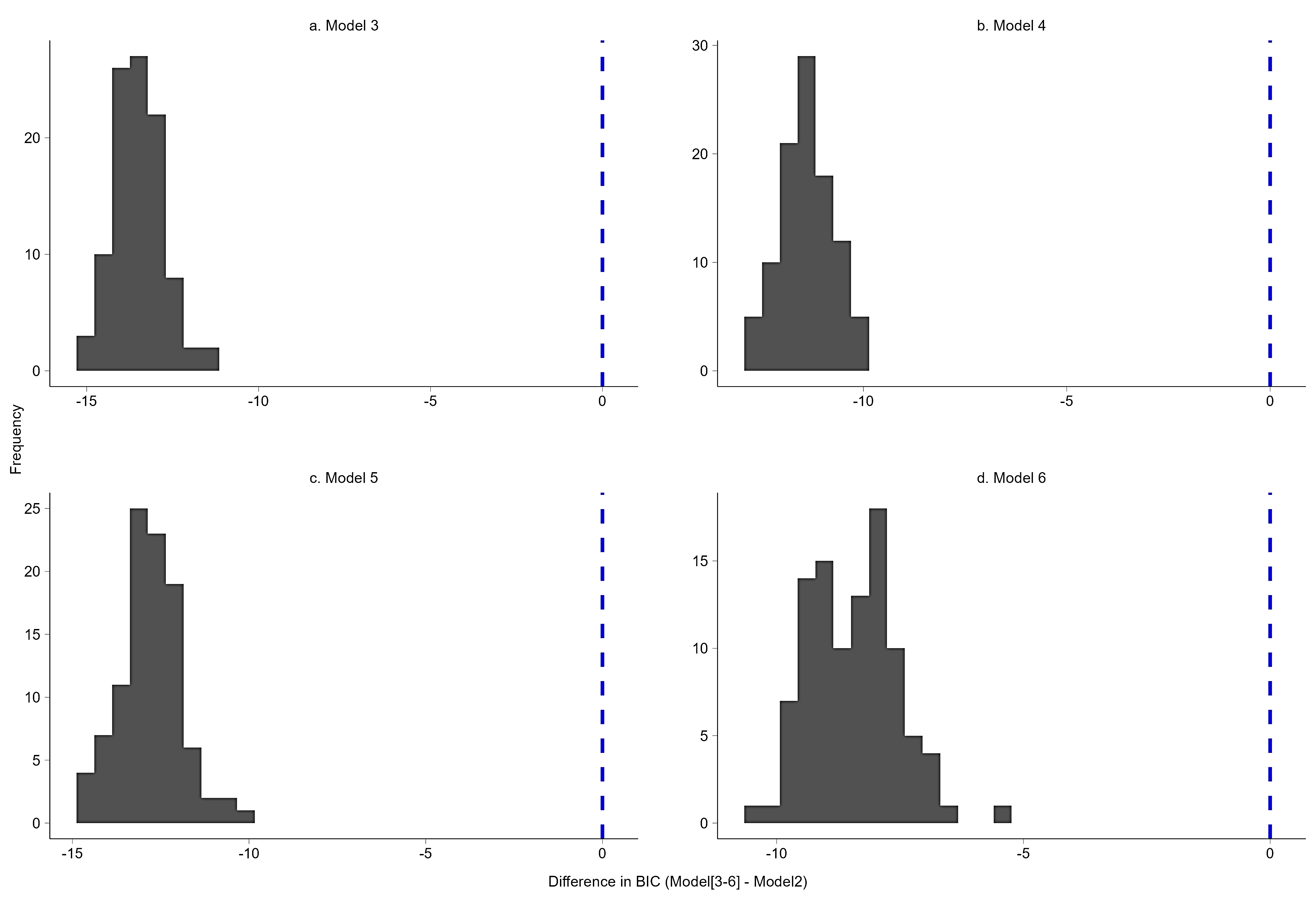


**Appendix V. Descriptions of exposure measures and their interpretation**

**Population density.** City population per square kilometers in all the urban patches inside the city geographic boundary as of 2010 or the closest year to 2010 for which official geographic boundaries were available. The population for each city was based on the projected population of all administrative units that comprise the city from official country estimates. Population is adjusted for United Nations’ country–level population projections. Urban patch definition is described below in Patch Density variable. The 2014 population density was used for this study.

**Patch density (Fragmentation).** The number of urban patches divided by the total area of a city (in 100 hectares) where an urban patch is a contiguous urban development. **Data sources:** To identify urban patches, 30 X 30m gridcells from the Global Urban Footprint (GUF) project from 2012 were used. For each built–up gridcell within the defined city boundaries, an algorithm classified them as urban if >50% of all gridcells within a one–km2 area surrounding it were built–up cells, suburban if 25–50% of gridcells were built–up and rural if <25% of gridcells were built–up. Urban clusters were then created based on contiguous urban, suburban and urbanized open space gridcells (e.g., a park surrounded entirely by built–up area). Urban clusters were then merged if adjacent clusters had contiguous or intersecting buffer areas 25% larger than the clusters. The resulting agglomerations, once no more merges were possible are the urban patches. The program FRAGSTATS 4.2 was used to calculate the number of urban patches per city. **Year of data:** 2012. **Interpretation:** Higher patch density reflects fragmentation of urban expansión.

**Area–weighted mean nearest neighbor (islolation).** Mean distance (in meters) to the nearest urban patch within the geographic boundary of the city. This value is weighted by the area of each patch. **Data sources:** The 2012 urban footprint data (in 30m x 30m grid cells) come from the ‘hole–filled” Global Urban Footprint (GUF). This variable is calculated based on 30m x 30m grid cells using the FRAGSTATS 4.2 software package. **Year of data**: 2012. **Interpretation:** Indicator of isolation among patches of a similar class, a higher value indicates higher level of isolation. By weighting by patch size, larger urban patches have a higher influence in this weighted–average metric.

**Intersection density.** The number of intersections (a point where more than 2 streets meet) per area of a city in square kilometers. **Data sources:** Intersections were extracted from street network OpenStreetMap data and included any intersections with >2 connected streets (i.e., cul–de–sacs and road bends represented as a node were excluded). Intersections per hectare. **Year of data:** September 2017. **Interpretation:** Varies from 0 to infinity. Cities with higher values have a lot of intersections by unit of area.

**Street length average.** The average length of street segments in meters (a section of a street between two intersections) in a city. **Data sources:** These data were extracted from OpenStreetMap data. **Year of data**: September 2017. **Interpretation:** Small values represent smaller blocks, and large values represent larger street segments or blocks.

**Streets per node average.** The average number of streets that meet at intersections in a city. **Data sources:** These data were extracted from OpenStreetMap data. **Year of data:** September 2017. **Interpretation:** Varies from 2 to infinity. Higher values mean on average intersections connect to more intersections.

**Average circuity.** Measures the average ratio of network distances to straight–line distances. **Data sources:** These data were extracted from OpenStreetMap data. **Year of data:** September 2017. **Interpretation:** For each edge in the network, its circuity is the ratio between the edge length and the straight-line distance between its two end points. Varies from 1 to infinity, where streets that are straight lines have circuity of 1, and curved streets have higher circuity.

**BRT, Subway or aerial tram Presence.** The presence or absence of a bus rapid transit (BRT), subway or aerial tram system in a city. **Data sources:** The data was collected from BRTData (<https://brtdata.org>) and OpenStreetMap. **Year of data:** September 2017.

**Urban Travel Delay Index.** Measures the increase in travel times due to congestion in the street network. Calculated as the average increase in travel times in a city imposed by congestion for 30 randomly selected pairs of points in the city and measured during seven points in time during peak traffic hours of a typical weekday. **Data sources:** The street network was obtained from OpenStreetMap and the travel times from Google Maps Distance Matrix API. **Year of data:** January 2018. **Interpretation:** Index ranges from zero to infinity. A value of zero means that traffic does not increase travel time. An index of X means travel time in traffic is (1+X) times the travel time without traffic.

**Social Environment Index.** This index comprises the proportion of households with piped water in their dwelling, proportion of dwellings with a sewage network connection, proportion of households with overcrowding (defined as more than three people per room; values were reversed for index calculation), and proportion of adults aged 25 years or older who completed at least primary education. **Data sources:** These measures are based on the most recent year of census data for the country: AR 2010; BR 2010; CL 2002; CO 2005; MX 2010; PA 2010 CR 2011. **Interpretation:** A higher score signifies a better social environment.

**Gross Domestic Product per Capita**. City–level GDP in 2010 in 2011 international US Dollars created by Genaioli et al in 2013 and converted into gridded estimates by Kummu et al in 2018. GDP for each year 1990–2015 was estimated by these researchers by modeling data from government, survey and industry. Gridded estimates were matched to SALURBAL cities and GDP was extracted directly from matching administrative units or using population–weighted averages if a city boundaries crossed multiple administrative areas. The 2014 GDP was used for this study.

Gennaioli N, La Porta R, Lopez–de–Silanes F, Shleifer A. Human capital and regional development. The Quarterly journal of economics. 2013;128(1):105–164. DOI: 10.1093/qje/qjs050

Kummu M, Taka M, Guillaume JHA. Gridded global datasets for Gross Domestic Product and Human Development Index over 1990–2015. Scientific data. 2018;5:180004. DOI: <https://doi.org/10.1038/sdata.2018.4>

**Registered motorcycles per 1,000 population.** Number of motorbike vehicles per city (this includes motorbikes, motorized tricycles and quadricycles and similar vehicles) / population* 1,000. Only available for Brazil, Chile, Colomba and Mexico. **Year of data:** 2014

**Appendix VI. Coefficient of models 1–2**.

RR were estimated using mixed effects generalized linear model regression with negative binomial distribution and robust standard errors. All RRs and 95% CIs reflect a difference of 1 standard deviation (SD) except as noted. All models are adjusted for a fixed effect for country, sex and 5–year age group. Model 1 is a single exposure model of each exposure and covariate. Model 2 includes all exposures and covariates in a multivariable model. Bolded values are statistically significant at P<0.05.

**Main analysis**

| **variable** | **Model 1 (95% CI)** | **Model 2 (95% CI)** |
| --- | --- | --- |
| Area–weighted mean nearest neighbour (islolation) | **1.12 (1.06–1.19)** | **1.07 (1.00–1.14)** |
| Average circuity | **0.93 (0.89–0.97)** | 0.97 (0.90–1.03) |
| Average street length | 1.03 (0.96–1.10) | 0.93 (0.87–1.01) |
| Average streets per node | **1.09 (1.03–1.14)** | 1.04 (0.97–1.11) |
| Gross Domestic Product per Capita (GDP) | 0.95 (0.89–1.02) | 0.98 (0.94–1.02) |
| Intersection density | **0.87 (0.83–0.91)** | **0.91 (0.83–0.99)** |
| Patch density (fragmentation) | **0.91 (0.86–0.95)** | 1.02 (0.96–1.10) |
| Population density in 2014 | **0.88 (0.82–0.94)** | **0.92 (0.85–1.00)** |
| Presence of bus rapid transit or subway (vs none) | **0.80 (0.75–0.86)** | 0.94 (0.87–1.03) |
| Social environment index | **0.84 (0.80–0.88)** | **0.88 (0.83–0.93)** |
| Urban travel delay index | **0.85 (0.80–0.90**) | 0.98 (0.91–1.05) |

**Subanalysis**

| **variable** | **Model 1 (95% CI)** | **Model 2 (95% CI)** |
| --- | --- | --- |
| Area–weighted mean nearest neighbour (islolation) | **1.13 (1.06–1.21)** | **1.08 (1.02–1.14)** |
| Average circuity | **0.94 (0.89–0.98)** | 0.99 (0.92–1.05) |
| Average street length | 1.04 (0.98–1.12) | 0.95 (0.88–1.03) |
| Average streets per node | **1.09 (1.04–1.14**) | 1.02 (0.96–1.09) |
| Gross Domestic Product per Capita (GDP) | 0.98 (0.93–1.04) | 0.99 (0.96–1.02) |
| Intersection density | **0.87 (0.83–0.91)** | 0.93 (0.86–1.01) |
| Patch density (fragmentation) | **0.91 (0.86–0.95**) | 1.01 (0.95–1.08) |
| Population density in 2014 | **0.87 (0.81–0.93)** | **0.92 (0.86–1.00)** |
| Presence of bus rapid transit or subway (vs none) | **0.81 (0.76–0.87**) | 0.97 (0.91–1.05) |
| **Registered motorcycles per 1,000 population** | **1.23 (1.08–1.41)** | **1.18 (1.04–1.34)** |
| Social environment index | **0.85 (0.81–0.89)** | **0.88 (0.84–0.93)** |
| Urban travel delay index | **0.84 (0.79–0.89)** | 0.98 (0.92–1.04) |
